# Metabolic Responses of Primary and Transformed Cells to Intracellular *Listeria monocytogenes*

**DOI:** 10.1371/journal.pone.0052378

**Published:** 2012-12-21

**Authors:** Nadine Gillmaier, Andreas Götz, Anette Schulz, Wolfgang Eisenreich, Werner Goebel

**Affiliations:** 1 Lehrstuhl für Biochemie, Technische Universität München, Garching, Germany; 2 Max-von-Pettenkofer Institut, Ludwig-Maximilians-Universität München, München, Germany; Aarhus University, Denmark

## Abstract

The metabolic response of host cells, in particular of primary mammalian cells, to bacterial infections is poorly understood. Here, we compare the carbon metabolism of primary mouse macrophages and of established J774A.1 cells upon *Listeria monocytogenes* infection using ^13^C-labelled glucose or glutamine as carbon tracers. The ^13^C-profiles of protein-derived amino acids from labelled host cells and intracellular *L. monocytogenes* identified active metabolic pathways in the different cell types. In the primary cells, infection with live *L. monocytogenes* increased glycolytic activity and enhanced flux of pyruvate into the TCA cycle via pyruvate dehydrogenase and pyruvate carboxylase, while in J774A.1 cells the already high glycolytic and glutaminolytic activities hardly changed upon infection. The carbon metabolism of intracellular *L. monocytogenes* was similar in both host cells. Taken together, the data suggest that efficient listerial replication in the cytosol of the host cells mainly depends on the glycolytic activity of the hosts.

## Introduction

Adaptation of the bacterial metabolism to their host cells is a crucial step in the replication cycle of intracellular bacterial pathogens. This important aspect of pathogenesis of intracellular bacteria has long been neglected, but has recently received increased attention (for recent reviews see [1], [2], [3], [4]). After internalization by suitable host cells (mainly dendritic cells, macrophages and epithelial cells), these bacteria are able to actively replicate either in specialized membrane-surrounded vacuoles, e.g. *Salmonella enterica, Legionella pneumophila, Mycobacterium tuberculosis, Chlamydia trachomatis, Brucella abortus*, or - after escape from the primary phagosome - in the host cell’s cytosol, e.g. *Shigella flexneri, Listeria monocytogenes,* and *Rickettsia prowazeki* [5], [6], [7]. Whereas the latter bacteria in principle have direct access to most nutrients taken up or produced by the host cells, the necessary basic nutrients must be first transported across the impermeable vacuolar membrane before they can reach vacuole-bound bacteria. Although research on intracellular bacterial metabolism is still in its infancy, the data already available suggest that each intracellular bacterial pathogen adapts its metabolism to preferred carbon and nitrogen sources offered by the host cells, which allows not only intracellular bacterial replication but seems to optimize also the expression of virulence factors essential for the intracellular life cycle [2], [3].

However, a possible flaw of these studies is that most of these data were obtained with established epithelial and macrophage cell lines. In contrast to the differentiated cells infected by the intracellular bacteria during *in vivo* infections, the established cell lines (used in the above mentioned studies) are cancer cells which carry out a significantly altered metabolism. Most normal cells use the tricarboxylic acid (TCA) cycle to produce ATP in the presence of oxygen by oxidative phosphorylation (OXPHO). Although OXPHO occurring in mitochondria provides more ATP than glycolysis, the glycolytic pathway can produce ATP at a higher rate [8]. The metabolism of cancer cells is subject to the “Warburg effect” [9] resulting in enhanced glucose uptake and preferential glucose catabolism via glycolysis even under normoxic conditions (“aerobic glycolysis”). Pyruvate, the end product of glycolysis, is converted to lactate under these conditions. Mitochondrial conversion of pyruvate to acetyl-CoA is often strongly suppressed and the metabolic flux through the TCA cycle, as well as aerobic respiration via the electron transport chain (ETC) is inhibited [10], [11]. Thus, ATP is mainly generated in cancer cells by enhanced glycolysis which favours - by increased glucose uptake - fast rather than efficient energy production.

In addition, enhanced glutaminolysis, i.e. uptake and conversion of glutamine to glutamate and further to α-ketoglutarate (α-KG) in the mitochondria, is also frequently observed in cancer cells and probably also in the established cell lines [12], [13]. Glutaminolysis feeds the mitochondrial TCA cycle leading to oxaloacetate (OAA) which together with glucose-derived acetyl-CoA results in enhanced citrate synthesis [14], [15]. Citrate can be translocated to the cytosol, where it is again broken down by ATP-dependent citrate lyase (ACL) to cytosolic acetyl-CoA and OAA necessary for the synthesis of fatty acids/lipids and amino acids (e.g. Asp), respectively. Under hypoxic conditions, however, this TCA-cycle dependent conversion of glutamine to citrate is strongly repressed due to the decreased formation of glucose-derived acetyl-CoA [16]. As compensation, increased citrate production by carboxylation of α-KG, catalyzed by mitochondrial isocitrate dehydrogenase 2 (IDH2) can occur [17].

Induction of “core” metabolic host cell genes may occur by the interaction with virtually all bacterial pathogens mainly via NF-κB activation, triggered by pathogen-associated molecular patterns (PAMPs) [18], [19] and by interferon-gamma (IFN-γ) [20], [21]. However, whether and how intracellular pathogens manipulate the host cell’s metabolism in a pathogen-specific fashion remains by and large an open, yet crucial question. The metabolism of mammalian cells is under the control of a complex regulatory network consisting of several signalling pathways that converge in the activation of several transcription factors, such as p53 [22], [23], Myc [24], [25] and HIF-1 [25], [26]. The three regulators control (among others) the expression of multiple genes involved in the uptake and metabolism of glucose and glutamine. These transcription factors, but also amino acid sensors like mTORC1 [27] and other nutrient “transceptors” [28], [29] controlling the host cell metabolism, and the constitutive expression and/or the altered activity of these regulatory components appear to be responsible for the metabolic deregulation of most cancer cells. These metabolic regulators may also represent potential host cell targets for the interaction with specific virulence factors and effector proteins of the bacterial pathogens and such interactions may lead to the reprogramming of the host cell metabolism.

As a first step to evaluate the need and efficiency of redistribution of resources between host cells and intracellular bacterial pathogens, we compared in this study the carbon assimilation of mouse bone marrow derived macrophages (BMM) and of macrophage-like J774A.1 cells without and with infection by *L. monocytogenes* using ^13^C-isotopologue profiling of amino acids. Primary host cells (like BMM) are of special interest in this context as they resemble more closely the target cells affected by *in vivo* infections than the commonly used established cell lines (like J774A.1). Recognition of specific metabolic host targets indispensible for the proliferation of intracellular bacterial pathogens may also provide novel approaches for antimicrobial interventions. The data indicate that the expectedly different core carbon metabolism of the two cell types is quite differently affected by intracellular *L. monocytogenes*. In sharp contrast, the carbon metabolism of the intracellular bacteria is similar in both host cell types.

## Results

### Experimental Approach for the Determination of the Basic Carbon Metabolism in Uninfected and *Listeria monocytogenes*-infected Primary and Transformed Murine Macrophages

^13^C-Isotopologue profiling of protein-derived amino acids is a powerful technique to determine active pathways and metabolite fluxes in living cells supplied with appropriate ^13^C-labelled carbon sources, such as glucose (reviewed in [3], [30]). To study possible differences in the metabolic responses of primary and established mammalian host cells to infection by intracellular bacterial pathogens, we applied this method to uninfected and *Listeria monocytogenes*-infected bone marrow macrophages derived from C57BL/6 mice (in the following abbreviated BMM) and macrophage-like J774A.1 cells derived from a murine reticulum sarcoma [31].

**Figure 1:**
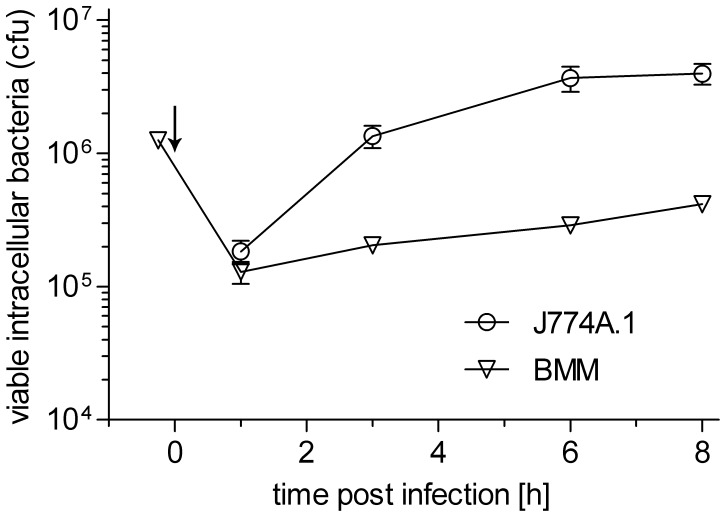
Growth of *L. monocytogenes* EGDe in BMM (triangles) and J774A.1 cells (circles ). Bacterial growth was determined over a period of 8 h post infection (pi). Indicated are the colony forming units (cfu) after infection of 2x10^5^ host cells with 5 bacteria per host cell. Each experiment was done three times in triplicate. Values are means ± SD of one representative experiment. The generation times given in the text were calculated from time point 1h pi to 8h pi for BMM and from 1h pi to 3h pi for J774A.1 host cells. Arrow indicates addition of gentamicin.

*Listeria monocytogenes* was taken up by both host cell types with similar efficiency (about 5% infected cells) and escape into the cytosol occurred at similar times (latest 1 h post infection, pi). The intracellular replication rates during the 8 h incubation period apparently differed considerably (Fig. 1). Moreover, at the time of harvest (8 h pi), the number of infected host cells increased up to 25% in J774A.1 cells, probably due to cell-to-cell spread of the intracellular listeriae, but not in BMM. This results in a 5-fold higher number of *L. monocytogenes*-infected J774A.1 cells (and accordingly more intracellular bacteria) in comparison with *L. monocytogenes*-infected BMMs. Since glucose and glutamine are the most important carbon sources for proliferating mammalian cells, uniformly ^13^C-labelled glucose ([U-^13^C_6_]glucose) or glutamine ([U-^13^C_5_]Gln) were used as precursors in experiments with uninfected and infected host cells. Separation of the intracellular bacteria and the host cells was performed as described previously [32]. In both fractions, the ^13^C-isotopologue profiles of *de novo*-synthesized (hence ^13^C-labelled) amino acids were determined after hydrolyzing protein under acidic conditions. Since the culture medium contained all amino acids, the biosynthesis of amino acids requiring multiple enzymatic steps should be repressed under these conditions. Indeed, ^13^C-incorporation occurred mainly into Ala, Glu and Asp from the host cells whose production requires only transamination of specific catabolic intermediates, generated by glycolysis (Ala from pyruvate) or by the TCA cycle (Asp from oxaloacetate (OAA) and Glu from α-ketoglutarate (α-KG)). Hence, the ^13^C-enrichments in Ala, Asp, and Glu reflect the cellular concentrations of the respective ^13^C-labelled catabolic intermediates and therefore allow conclusions concerning the activity of the metabolic pathways leading to these intermediates in the uninfected and infected host cells.

Comparing the labelling profiles of amino acids from infected and uninfected host cells, metabolic changes caused by the intracellular cytosolically replicating *L. monocytogenes* could be identified. To specifiy the impacts due to live *L. monocytogenes*, ^13^C-profiles of host cells treated with UV-inactivated *L. monocytogenes* or interferon-gamma (IFN-γ) [33] were also determined.

### ^13^C-Profiles of Amino Acids from BMM Cells Cultured in the Presence of [U-^13^C_6_]Glucose

**Figure 2:**
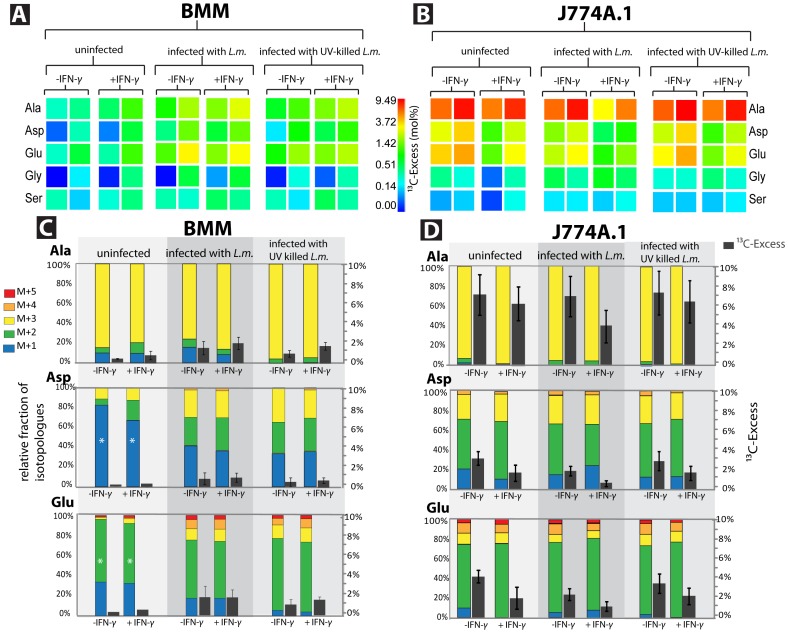
^13^C-Profiles of amino acids from host cells cultivated in the presence of [U-^13^C_6_]glucose. (A) ^13^C-Excess (mol %; displayed as a color code) of amino acids derived from BMM cells; for each condition, two independent labeling experiments were performed. The color for each amino acid correlates with the mean value of three MS determinations with the same sample. (B) ^13^C-Excess (mol %; displayed as a color code) of amino acids derived from J774A.1 cells; for each condition, two independent labeling experiments were performed. The color for each amino acid correlates with the mean value of three MS determinations with the same sample. (C) Relative isotopologue composition of amino acids derived from BMM. Colored columns indicate the % amounts of isotopologues comprising 1, 2, 3, 4, or 5 ^13^C-atoms (M+1 to M+5; left scale; * indicate values from only one labeling experiment; otherwise, the values represent means from two labeling experiments). ^13^C-Excess values are shown as grey columns (mol %, right scale; error bars indicate SD for two independent labeling experiments). (D) Relative isotopologue composition of amino acids derived from J774A.1 cells. Colored columns indicate the % amounts of isotopologues comprising 1, 2, 3, 4, or 5 ^13^C-atoms (M+1 to M+5; left scale; the values represent means from two labeling experiments). ^13^C-Excess values are shown as grey columns (mol %, right scale; means from two independent labeling experiments; error bars indicate SD for the two labeling experiments). Experiments were done with and without infection by *Listeria monocytogenes* (*L.m.*) and with (+) and without (-) activation by interferon-γ (IFN-γ). The color maps indicate ^13^C-excess in semi-logarithmic form to show even relatively small ^13^C excess values; for numerical values, see also Table S1.

In uninfected BMM cells, low ^13^C-incorporation occurred mainly into Glu and Ala, when fed with [U-^13^C_6_]glucose for 8 h (Fig. 2A). Under these conditions, [^13^C_3_]-Ala, [^13^C_1_]-Asp, [^13^C_1_]- and [^13^C_2_]-Glu were predominantly formed (Fig. 2C), suggesting that [U-^13^C_6_]glucose is converted at a low rate via the glycolytic pathway to [^13^C_3_]-pyruvate (the precursor of [^13^C_3_]-Ala), which is further degraded by pyruvate dehydrogenase (PDH) to ^13^CO_2_ and [^13^C_2_]-acetyl-CoA. The latter metabolite is channeled into the TCA cycle and linked to unlabelled oxaloacetate (OAA – for example derived from unlabelled Gln - see below) yielding [^13^C_2_]-α-KG, the precursor of [^13^C_2_]-Glu (flux 1 in Fig. S1). Formation of the [^13^C_1_]-isotopologues can be explained by carbon flux via pyruvate carboxylase (PYC) involving ^13^CO_2_ as a substrate (flux 2 in Fig. S1).

Infection with live (and at a lower extent by UV-inactivated) *L. monocytogenes* not only caused enhanced ^13^C-incorporation into Ala, Asp and Glu (Fig. 2A), but also changed the isotopologue pattern of Glu. Besides the major [^13^C_2_]-Glu species (formation via flux 1 in Fig. S1), [^13^C_1_]-, [^13^C_3_]-, [^13^C_4_]-, and even [^13^C_5_]-Glu were now also formed albeit in lower amounts (Fig. 2C). The ^13^C-α-KG precursors required for these latter Glu isotopomers can be generated by PYC- and PDH-catalyzed reactions with different combinations of labelled and unlabelled pyruvate and CO_2_ leading to [^13^C_1_]-, [^13^C_3_]-, [^13^C_4_]-OAA, which in combination with [^13^C_2_]-labelled and unlabelled acetyl-CoA (derived from unlabelled Gln - see below) result in the formation of the [^13^C_1_]-, [^13^C_3_]-, [^13^C_4_]-, and [^13^C_5_]-α-KG precursors (flux 2 in Fig. S1).

Under infection conditions, similar amounts of [^13^C_1_]-, [^13^C_2_]-, and [^13^C_3_]-Asp isotopologues were formed. The [^13^C_1_]- and [^13^C_3_]-Asp isotopologues require as precursors [^13^C_1_]- and [^13^C_3_]-OAA, respectively, which are generated by ATP-dependent pyruvate carboxylase (PYC) either from unlabelled pyruvate (derived from unlabelled Gln - see below) and ^13^CO_2_ or from [^13^C_3_]-pyruvate and unlabelled CO_2_ (flux 2 in Fig. S1). ^13^CO_2_ could originate either from the ^13^C-labelled pyruvate by PDH or from decarboxylation of [^13^C_6_]-6-phosphogluconate in the pentose phosphate pathway. The [^13^C_2_]-OAA precursor for [^13^C_2_]-Asp is formed in the TCA cycle by linking [^13^C_2_]-acetyl-CoA to unlabelled OAA (again derived from Gln - see below) (flux 1 in Fig. S1). In IFN-γ-activated BMM, ^13^C-incorporation into Glu, Asp and Ala slightly increased as compared to untreated BMM (Fig. 2A).

The very weakly ^13^C-labelled amino acids Ser and Gly showed the expected [^13^C_3_]-Ser and [^13^C_2_]-Gly isotopologues, deriving from the glycolytic intermediate [^13^C_3_]-3-phosphoglycerate. Neither infection of BMM by *L. monocytogenes* nor IFN-γ activation seem to change the rate of ^13^C-incorporation into the latter amino acids in BMM.

Together, the data show that BMM cells exhibit low metabolic activity, but viable *L. monocytogenes* (and to a lesser extent IFN-γ and UV-inactivated bacteria) enhance glycolysis leading to an increased level of labelled pyruvate, which is channeled at increased rates into the TCA cycle via the PDH- and PYC-generated intermediates.

### ^13^C-Profiles of Amino Acids from J774A.1 Cells Cultured in the Presence of [U-^13^C_6_]Glucose

Uninfected J774A.1 cells grown in the presence of [U-^13^C_6_]glucose showed much higher ^13^C-incorporation into Glu, Ala and Asp (up to 5-fold depending on the amino acid) than BMM (Fig. 2B). ^13^C-Incorporation into Glu, Asp and Ala significantly decreased in IFN-γ-treated cells and slightly decreased upon infection with live *L. monocytogenes*, but not with UV-inactivated *L. monocytogenes* (Fig. 2B). This is in sharp contrast to ^13^C-incorporation into the same amino acids from BMM host cells, where infection with *L. monocytogenes* leads to a considerable increase. This suggests the presence of a highly active glucose-derived metabolite flux through the glycolytic pathway already in the uninfected J774A.1 cells and at least through those steps of the TCA cycle, which are necessary for the generation of OAA and α-KG, the intermediates for Asp and Glu. This flux is - in contrast to BMM - not further activated by the *L. monocytogenes* infection. The ^13^C-incorporation into Ser and Gly from uninfected and infected J774A.1 cells was as low as in BMM cells (Fig. 2B).

Other than in BMM, the isotopologue distribution of J774A.1 amino acids did not significantly change upon infection with live or killed *L. monocytogenes* or by treatment with IFN-γ. Notably, the ^13^C-isotopologue patterns of Ala, Asp and Glu from uninfected J774A.1 were quite similar to those from *L. monocytogenes*-infected BMM cells (Fig. 2D), arguing for the generation of these isotopologues by similar pathways in both cases (see above and Fig. S1). Thus, the high level of [^13^C_3_]-Ala (deriving from [^13^C_3_]-pyruvate) indicates a high metabolite flux of the added [U-^13^C_6_]glucose through the glycolytic pathway in J774A.1 cells. The low amount of ^13^C-labelled Ser and Gly apparently derived from the glycolytic intermediate [^13^C_3_]-3-phosphoglycerate. These data again show that biosynthesis of amino acids that requires more than only transamination of a glycolytic or TCA intermediate is repressed when the corresponding amino acid are externally supplied, regardless whether the carbon metabolism is low (as in BMM) or high (as in J774A.1).

Additionally and in contrast to the experiments with BMMs, lower^ 13^C-incorporation into the above mentioned amino acids was observed when IFN-γ activated J774A.1 cells were used, indicating that IFN-γ activation has a different effect on carbon metabolism in the J774A.1 tumor cell line.

In summary, the results show that the glycolytic pathway and at least the α-KG- and OAA-producing steps of the TCA cycle which are also essential for anabolic processes (amino acid, nucleotide and lipid biosynthesis) are highly active in J774A.1 cells. *L. monocytogenes* infection hardly alters these metabolic steps.

### ^13^C-Profiles of Amino Acids from BMM and J774A.1 Cells Cultured in the Presence of [U-^13^C_5_]Glutamine

**Figure 3:**
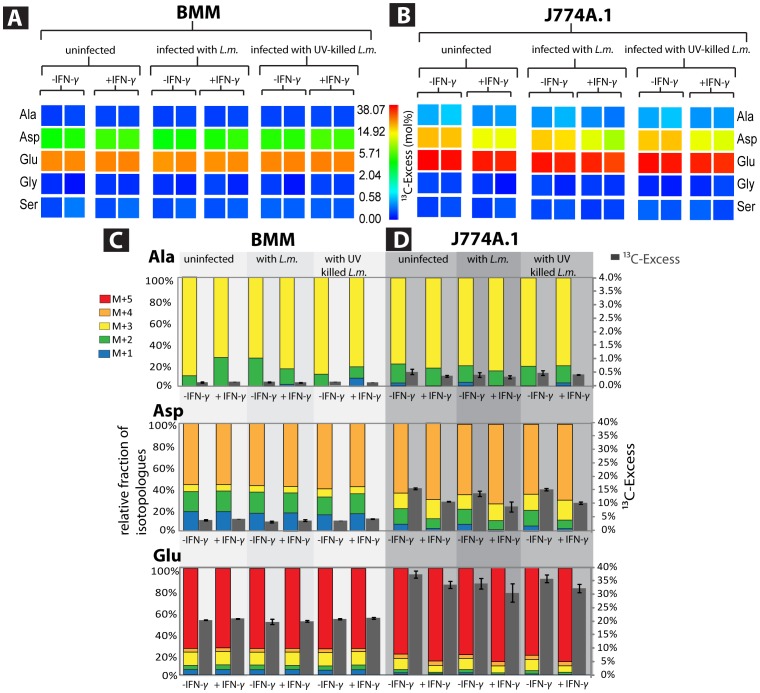
^13^C-Profiles of amino acids from host cells cultivated in the presence of [U-^13^C_5_]glutamine. (A) ^13^C-Excess (mol %) of amino acids derived from BMM cells, (B) ^13^C-Excess (mol %) of amino acids derived from J774A.1 cells, (C) Relative isotopologue composition of amino acids derived from BMM. (D) Relative isotopologue composition of amino acids derived from J774A.1 cells. For more details, see legend to Figure 2; for numerical values, see also Table S2.

Next, BMM and J774A.1 cells without or with infection by *L. monocytogenes* were supplemented with 2 mM [U-^13^C_5_]Gln. In both host cell types, the major amount of ^13^C-label was found - as expected - in [U-^13^C_5_]Glu, indicating that most of the supplied [U-^13^C_5_]Gln was directly incorporated into protein (Fig. 3). Substantial ^13^C-incorporation was also found in Asp indicating that Gln is in part used for replenishing the TCA cycle to support anabolic reactions in uninfected and infected cells. Incorporation into Asp from J774A.1 cells was about 3 to 5-fold higher compared to incorporation into Asp from BMM cells (Fig. 3A and B). It can be concluded that the anaplerotic reactions via glutaminolysis were more active in J774A.1 cells.

In addition to the major [^13^C_5_]-Glu isotopologue, mainly [^13^C_3_]-Glu and in small amounts also [^13^C_1_]- and [^13^C_2_]-Glu were generated in BMM, as well as in J774A.1 cells. ^13^C-Asp isotopologues were formed in the order [^13^C_4_]>[^13^C_1_] = [^13^C_2_]>[^13^C_3_] in BMM, but in the order [^13^C_4_]>[^13^C_3_]>[^13^C_2_]>[^13^C_1_] in J774A.1 cells, suggesting that some metabolic reactions were differently activated in the two cell types. On the other hand, the ^13^C-isotopologue profiles of these amino acids were not significantly changed by the *L. monocytogenes* infection (Figs. 3C and 3D). Activation by IFN-γ caused a slight reduction (about 30%) of ^13^C-incorporation in viable J774A.1 cells, but not in BMMs.

Obviously, [^13^C_5_]-Gln is transported into the mitochondria where it is converted to [^13^C_5_]-α-KG, [^13^C_4_]-succinate, [^13^C_4_]-fumarate, [^13^C_4_]-malate, [^13^C_4_]-OAA and ^13^CO_2_ in the TCA cycle, running in the canonical direction (flux 1 in Fig. S2). Unlabelled acetyl-CoA (deriving from the unlabelled glucose via glycolysis) when linked to [^13^C_4_]-OAA yields [^13^C_3_]-α-KG. Indeed, this pathway leads to the major ^13^C-isotopologues of Glu ([^13^C_5_] and [^13^C_3_]) and Asp ([^13^C_4_]), suggesting that it is mainly used in BMM and J774A.1 cells. The fluxes 2, 3 and 4 in Fig. S2 explain the formation of the other (minor)^ 13^C isotopologues of Glu and Asp. Of special interest is the generation of [^13^C_3_]-Asp, which is the second major ^13^C-Asp isotopologue in J774A.1, but not in BMM. This Asp isotopologue can be mainly generated by flux 2 shown in Fig. S2, which is initiated by the reductive carboxylation of α-KG to isocitrate catalyzed by NADPH-dependent mitochondrial isocitrate dehydrogenase (IDH2). This reverse IDH2 reaction has been demonstrated in cancer cells [17], [34] and obviously also occurs in J774A.1 cells.

Small, but well reproducible ^13^C-label was also observed in Ala, Ser and Gly from both cell types (for numerical values, see Table S1). These ^13^C-isotopologues were probably formed from intermediates generated by gluconeogenesis starting from [^13^C_4_]-OAA (flux 1). Infection by *L. monocytogenes* again hardly changed the rates of ^13^C-incorporation into these amino acids in the two cell types.

In summary, glutaminolytic activity in BMM cells is similarly low as the glycolytic activity. Flux through glutaminolysis remains low upon *L. monocytogenes* infection. In contrast, glutaminolysis is much more active in J774A.1 cells and might be even slightly inhibited by the *L. monocytogenes* infection.

### ^13^C-Profiles of Amino Acids from *L. monocytogenes* Replicating in BMM or J774A.1 Cells

**Figure 4:**
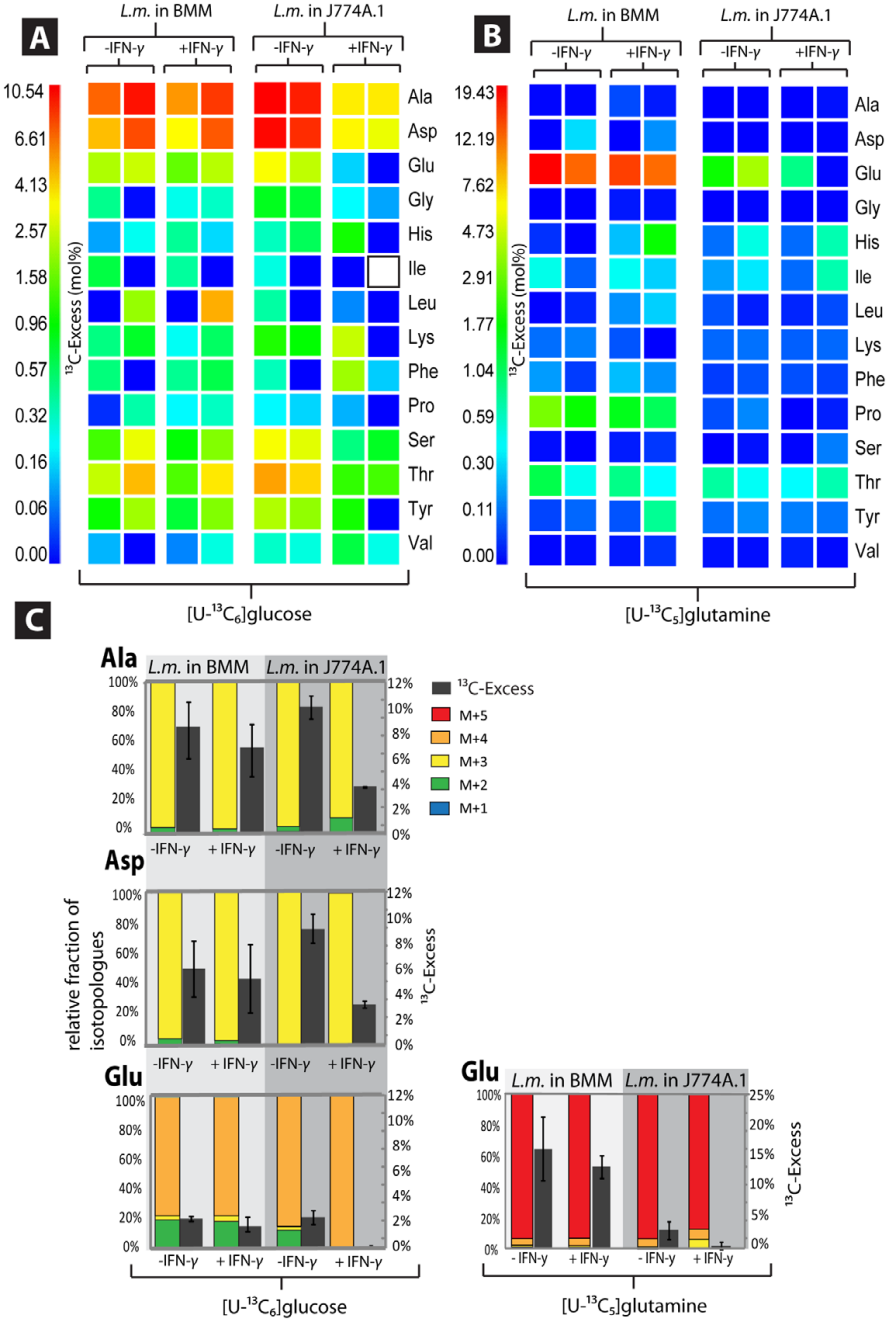
^13^C-Profiles of amino acids from *Listeria monocytogenes* replicating in BMM or J774A.1 host cells. (A) Experiment with [U-^13^C_6_]glucose. ^13^C-Excess (mol %) displayed as a color map. (B) Experiment with [U-^13^C_5_]glutamine.^ 13^C-Excess (mol %) displayed as a color map. (C) Relative isotopologue compositions. Colored columns indicate the % amounts of isotopologues comprising 1, 2, 3, 4, or 5 ^13^C-atoms (M+1 to M+5; left scale). ^13^C-Excess values are shown as grey columns (mol %, right scale); for numerical values, see also Tables S1 and S2.

**Figure 5:**
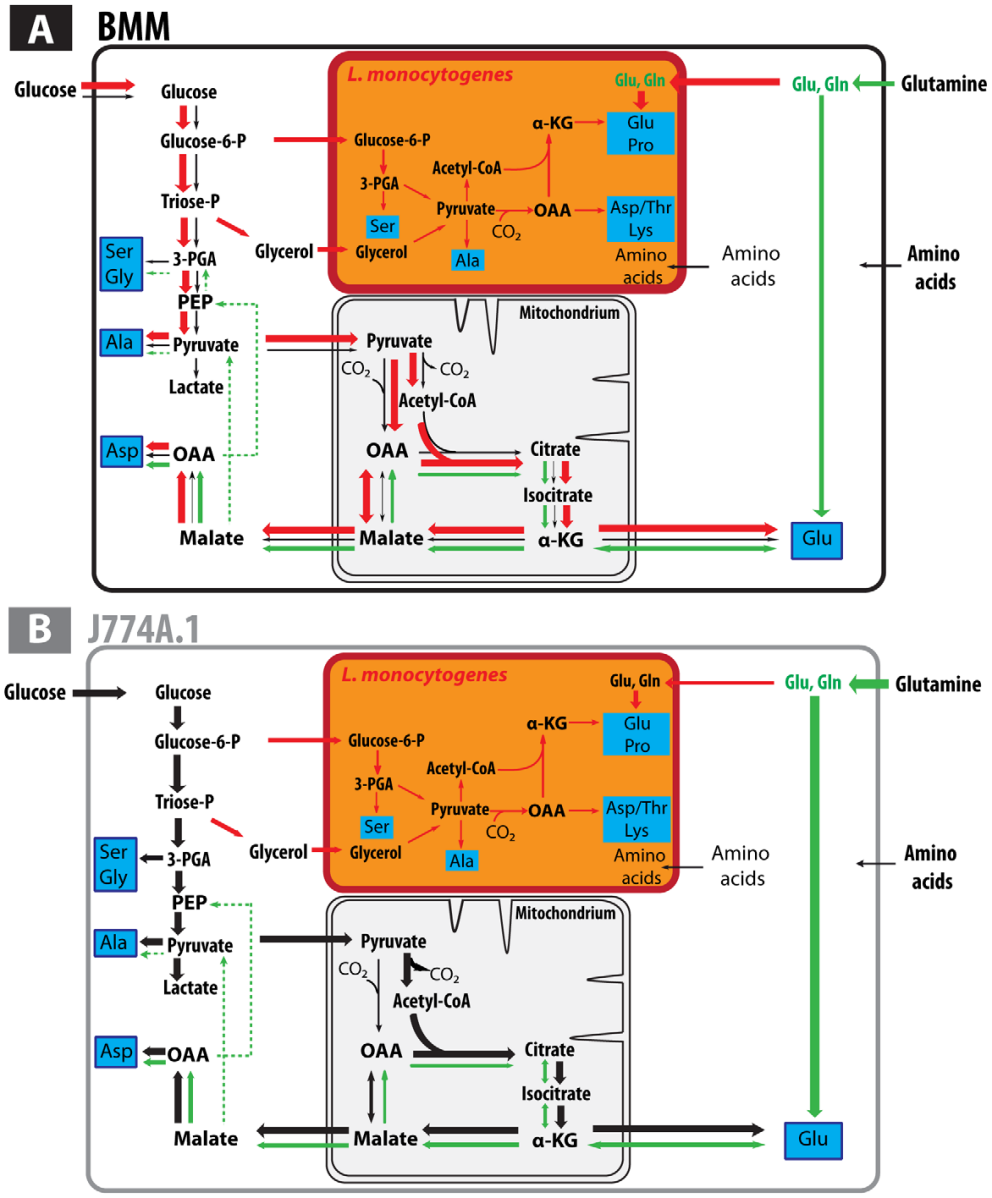
Reconstructed metabolite fluxes originating from glucose or glutamine in BMM (A) and J774A.1 cells (B) with or without infection by *L. monocytogenes*. Black arrows indicate glucose-derived fluxes. Line widths indicate the approximate activities through these reactions. Green arrows indicate fluxes via glutaminolysis. Red arrows display flux modulations due to infection with *L. monocytogenes*. Bacterial pathways are highlighted in orange. The listerial metabolism is mainly fed by host cell derived [U-^13^C_3_]glycerol (deriving from [U-^13^C_6_]glucose) and [U-^13^C_6_]glucose 6-phosphate as described before [32]. The ^13^C-labelled intermediates for the formation of the major ^13^C-amino acid isotopologues derive from glycolytic reactions to pyruvate which is converted by PDH to Ac-CoA. The latter intermediate is channeled into the TCA cycle yielding together with OAA citrate and further to α-KG. OAA is produced by PYC-catalyzed carboxylation of pyruvate due to the disrupted listerial TCA cycle [47] which is also the reason for the inability of *L. monocytogenes* to catabolize [U-^13^C_5_]glutamine.

Regardless whether the bacteria replicated in BMM or J774A.1 cells, the same bacterial amino acids (in the order Ala>Asp>Thr>Glu>Ser>Tyr>Lys>Gly) were ^13^C-labelled in the presence of [U-^13^C_6_]glucose (Fig. 4A, for numerical values, see Table S2). Even the absolute ^13^C-enrichment values and the isotopologue patterns (Fig. 4C) were similar in amino acids from *L. monocytogenes*, irrespective of growth in BMM or J774A.1 cells. This indicates that in both host cells the same glucose-derived carbon sources were used and metabolized in a similar way by intracellular *L. monocytogenes* (see key reactions highlighted in orange in Fig. 5). In IFN-γ-treated BMM cells, no change of the rate of ^13^C-incorporation into the amino acids and of the ^13^C-isotopologue patterns was observed, whereas a 2-fold reduction in the rate of ^13^C-incorporation into the amino acids occurred in IFN-γ-treated J774A.1 cells (Fig. 4A).

In the presence of [U-^13^C_5_]-Gln, *L. monocytogenes* replicating in BMM incorporated substantial amounts of [^13^C_5_]-Glu/Gln into bacterial protein (Figs. 4B and 4C), but apparently did not metabolize [^13^C_5_]-Glu/Gln further, since no other amino acid, except Pro which derives directly from Glu, acquired significant ^13^C-label. This is not unexpected, since *L. monocytogenes* lacks α-ketoglutarate dehydrogenase which should be necessary for channeling [^13^C_5_]-Glu/Gln as carbon source into the listerial TCA cycle. *L. monocytogenes* replicating in J774A.1 incorporated considerably less [^13^C_5_]-Glu/Gln into protein (Fig. 4B). IFN-γ inhibited the formation of [^13^C_5_]-Glu/Gln in *L. monocytogenes* when the bacteria replicated in J774A.1 cells, but not when they replicated in BMM cells (Figs. 4 B and 4C).

These data show that the carbon metabolism of intracellularly growing *L. monocytogenes* is similar in BMM and J774A.1 and mainly depends on the glycolytic activity of the host cells, while the glutaminolytic activity does not seem to affect the listerial carbon metabolism.

## Discussion

Glucose and glutamine are the major carbon sources providing energy and intermediates for anabolic processes to proliferating mammalian cells via glycolysis, the pentose phosphate shunt, glutaminolysis and the TCA cycle. ^13^C-Isotopologue profiling using [U-^13^C_6_]glucose or [U-^13^C_5_]glutamine was applied to determine the carbon pathways in primary murine bone marrow macrophages (BMM) and the established murine macrophage cell line J774A.1. The activities in the core carbon metabolism of the two cell types turned out to be quite different. Glycolytic and glutaminolytic activities were low in BMMs, but high in neoplastic J774A.1 macrophages (cf. line widths of black and green arrows in Fig. 5), which is a typical feature (“Warburg effect “) of many cancer cells [11], [13].

The enhanced metabolic activities in J774A.1 cells can be explained by the fact that this cell line derives from a mouse tumor expressing c-Myc constitutively [35]. This transcription factor causes up-regulation of the genes for the glucose transporter GLUT1, several glycolytic enzymes and lactate dehydrogenase (LDH-A) [25], [36], [37]. Myc also induces a transcriptional program that promotes glutaminolysis by increasing the level of a glutamine transporter and mitochondrial glutaminase (GLS) [38], leading to cell adaptation to glutamine as a bioenergetic substrate. Indeed, the carbon metabolism of J774A.1 cells, deduced from the ^13^C-profiles of Ala, Glu and Asp, displayed the expected Myc-induced glycolytic and glutaminolytic pathways [23], [24]. Interestingly, substantial reductive carboxylation of glutamine-derived α-KG to citrate, catalyzed by the mitochondrial IDH2, occurred in J774A.1 cells, but not in BMM under any of the described experimental conditions. This reaction, running in the opposite direction to the canonical TCA cycle has also been previously observed in cancer cells [34]. It generates citrate for anaplerosis under hypoxic conditions and appears to be HIF-1-regulated under these conditions [17].

Interestingly, the metabolic patterns in J774A.1 were hardly altered by infection with live (or killed) *L. monocytogenes*, suggesting that the carbon metabolism of J774A.1 cells is already appropriate for efficient intracellular metabolism of *L. monocytogenes*. IFN-γ led to an apparent reduction of the glycolytic and the glutaminolytic activities which may be due to enhanced production of reactive oxygen species (ROS) causing reduced viability of these host cells [39].

In sharp contrast, infected BMM cells showed a strongly increased glycolytic activity (red arrows in Fig. 5) in comparison with uninfected BMM cells, whereas carbon flux via glutaminolysis remained low. Taking into consideration the relatively low portion of infected cells in the *L. monocytogenes*-treated BMM population (see above), the increment in metabolic induction caused by the intracellular *L. monocytogenes* should be even higher than expressed by the reported experimental numbers. Activation by IFN-γ or UV-killed *L. monocytogenes* induced a similar flux modulation, albeit at a considerably lower level.

The IFN-γ-mediated relatively low induction may be due to enhanced expression of inducible nitric oxide synthase (iNOS or NOS2) [40], [41]. The thereby increased amount of NO activates glucose uptake and glycolysis in macrophages and other cells [42]. Similar iNOS induction has also been shown for live and killed bacteria by PAMP-induced NF-κB activation [19] and may explain the slight increase of metabolic activity by UV-inactivated bacteria. The high induction of glycolysis in BMM observed with live *L. monocytogenes* requires, however, not only uptake of the listeriae (which is similar for the live and UV-inactivated *L. monocytogenes*), but also release of the bacteria into the host cell’s cytosol (which occurs with live but not with UV-inactivated *L. monocytogenes*). The thereby triggered induction of host cell glycolysis could be caused by HIF-1 activation (via stabilization of the HIF-1α subunit) and is indeed frequently observed during infections with extra- and intracellular bacterial pathogens [43], [44]. However, activated HIF-1 blocks PDH activity (through activation of PDH kinase, [45]) and thus inhibits the TCA cycle. On the other hand, the induced *de novo* synthesis of Glu and Asp in BMM (particularly when infected by live *L. monocytogenes*) requires active pyruvate dehydrogenase (PDH), pyruvate carboxylase (PYC), citrate synthase (CS), isocitrate dehydrogenase (probably IDH1 and IDH2), ATP-dependent citrate lyase (ACL) and malic enzyme (ME). Hence, PDH must be active in the infected BMMs which becomes particularly evident by the increased production of [^13^C_2_]-Glu as major ^13^C-Glu isotopologue species in the infected BMM cells when cultured in the presence of [U-^13^C_6_]glucose. Generation of this Glu isotopologue requires [^13^C_2_]-acetyl-CoA that can only be produced by PDH from [U-^13^C_6_]glucose-derived [^13^C_3_]-pyruvate. Thus, HIF-1 is probably not responsible for the highly induced glycolysis in BMMs when infected with live *L. monocytogenes* and it remains an intriguing question which host cell regulator is responsible for this induction and which listerial factor triggers it. Listeriolysin might be a candidate on the listerial side [46].

Notably, the carbon metabolism of the intracellular *L. monocytogenes*, deduced from the ^13^C-profiles of the labelled amino acids by [U-^13^C_6_]glucose, was similar in BMM and J774A.1. Substantial ^13^C-incorporation did not only occur into bacterial Ala, Glu and Asp, but also into Ser, Gly, Pro, Thr and Lys, indicating that the intracellular listeriae sense the obviously low level of these latter amino acids in the host cell cytosol, whereas the host cells sense, as expected, the high external amino acid concentrations. The bacterial carbon metabolism is apparently not influenced by the highly different efficiency of glutamine uptake and consumption by the two host cell types. This may be due to the missing α-ketoglutarate dehydrogenase [47] and the inefficient uptake of OAA and Asp [48] into *L. monocytogenes,* rendering its catabolism insensitive to glutamine. As shown previously [32], intracellular *L. monocytogenes* seem to prefer glycerol and glucose 6-phosphate as carbon sources provided by the host cell. Indeed, the availability of these carbon compounds depends only on the glycolytic but not on the glutaminolytic activity of host cells.

In summary, the data show that *L. monocytogenes* infections modifies the metabolism of primary host cells (at least of BMM) with an *a priori* inadequate metabolism for listerial replication in such a way that it becomes appropriate for the intracellular metabolism of these bacteria.

## Materials and Methods

### Bacterial Strains, Macrophage Cells and Growth Media

*L. monocytogenes* strain EGDe and the used murine macrophage-like cell line J774A.1 were described before [32]. Primary C57BL/6 mouse macrophages were obtained from the bone marrow and differentiated to CD11b+, CD11c-, F4/80+ macrophages in Dulbecco's Modified Eagle Medium (DMEM) containing 4.5 g/l glucose, 110 mg/l sodium pyruvate and supplemented with 2 mM glutamine, 10 mM HEPES, 10 mg/l recombinant murine macrophage colony-stimulating factor (M-CSF), 10% FCS and 5% horse serum for 8 to 10 days; fresh medium was added every 3 days. Mice were cared for in accordance with the principles outlined by the European Convention for the Protection of Vertebrate Animals Used for Experimental and Other Scientific Purposes (European Treaty Series, no. 123; http://conventions.coe.int/Treaty/en/Treaties/Html/123). All animal experiments were in compliance with the German animal protection law and were approved (permit no. 03-001/08) by the responsible Federal State authority and ethics committee. The isolation and use of mouse macrophages were approved by the responsible Federal State authority (AZ 55.2-1-54-2531.6-14-02).

### Growth Conditions and Infection

*L. monocytogenes* was grown in BHI at 37°C with aeritation to an OD_600_ ≈ 1. Then, the culture was centrifuged at 7,000 × *g* and washed two times with ice-cold PBS. Aliquots resuspended in PBS containing 20% (v/v) glycerol were frozen at -80°C and used for later infection. The differentiated mouse primary macrophages (BMM) and the J774A.1 cells were seeded in 24 hole plates at cell densities of 2×10^5^ and 1×10^5^ cells per hole, respectively, and incubated overnight to allow adherence of the macrophages. BMM and J774A.1 cells were infected with *L. monocytogenes* EGDe (5 bacteria per host cell) resuspended in RPMI medium with 10% FCS and 10 µM recombinant human insulin to enhance glucose uptake by BMM [49]. Infection was synchronized by centrifugation at 173 × *g* and 37°C for 5 min. After 60 min of incubation, the medium was replaced by RPMI containing additionally 50 µg/ml gentamicin to kill extracellular bacteria. This was defined as time point 0 post infection (pi). Under these conditions, up to 5% of the BMM and J774A.1 cells contained intracellular bacteria. Intracellular bacterial growth was measured over a period of 8 h by lysing the cells in distilled water and counting the released viable bacteria by plating them on BHI agar plates.

### ^13^C-Labelling Experiments

2×10^6^ BMM and 1×10^6^ J774A.1 cells, respectively, were seeded in culture plates (Ø 10 cm), containing medium supplemented with unlabelled substrates, 20 ng/ml interferon-gamma (IFN-γ) where indicated, and incubated for 24 h. Infection was carried out as described above with *L. monocytogenes* EGDe at a MOI of 5. As controls, an equal number of host cells remained uninfected or were infected with UV-inactivated *L. monocytogenes* (5 bacteria per host cell). After 30 min of gentamicin-treatment, the medium was replaced by fresh RPMI now containing additionally 10 mM [U-^13^C_6_]glucose (without unlabelled glucose, but 2 mM unlabelled glutamine) or 2 mM [U-^13^C_5_]glutamine (no unlabelled glutamine, but 10 mM unlabelled glucose). At 8.5 h pi, cells were washed with PBS buffer and lysed in 1 ml ice-cold distilled water containing 0.1% TritonX-100, 10 mM sodium azide, 5 µg/ml tetracycline and 50 µg/ml chloramphenicol for 10 min. The lysate was vigorously vortexed and the bacteria were pelleted at 25,000 × *g*. The bacteria-free supernatant was frozen (in liquid nitrogen). The bacterial pellet was washed with RIPA buffer [50], recentrifuged and frozen in liquid nitrogen without resuspending it.

### Protein Hydrolysis and Amino Acid Derivatization

Protein-containing samples (i.e. bacteria-free supernatant or bacterial pellet, see above) were heated in 6 M HCl for 24 h at 105°C. Under these conditions, protein-derived Gln and Asn were converted into Glu and Asp, respectively. Trp and Cys were destroyed by this treatment. HCl was evaporated under nitrogen and the pellet was dissolved in 50% acetic acid. Separation and purification of the protein-derived amino acids was performed via a cation exchange column of Dowex 50Wx8 (H^+^-form, 200–400 mesh, 5×100 mm), washed with water and developed with 4 M ammonium hydroxide. The eluate was dried under nitrogen, and the residue dissolved in 50 µl water-free acetonitrile. A mixture of 50 µl of *N*-(*tert*-butyldimethylsilyl)-*N*-methyl-trifluoroacetamide containing 1% *tert*-butyldimethylsilylchloride (Sigma) was added. The derivatization took place at 70°C for 30 min. The resulting N-(tert-butyldimethylsilyl) (TBDMS)-amino acids were then measured via GC/MS. The yields of TBDMS-Arg and TBDMS-Met were low. Therefore, isotopologue data of theses amino acids are typically not listed.

### Gas Chromatography/mass Spectrometry (GC/MS)

GC/MS was performed on a GC 2010 (Shimadzu, Duisburg, Germany) equipped with a fused silica capillary column (Equity TM-5; 30×0.25 µm film thickness; SUPELCO, Bellefonte, PA) and a quadrupol QP2010 plus detector (Shimadzu) working with electron impact ionization at 70 eV. The derivatized sample was injected in a 1∶10 split mode at an interface temperature of 260°C and a helium inlet pressure of 70 kPa. The GC-column was developed at 150°C for 3 min and then with a temperature gradient of 7°C/min to a final temperature of 280°C which was held for 3 min. With a sampling rate of 0.5 s, selected ion monitoring was used. Data collection proceeded via GC/MS Solution software (Shimadzu). All samples were measured three times. ^13^C-Excess and isotopologue abundances were calculated according to standard procedures [51], [52] including: (i) determination of the TBDMS-derivate spectrum of derivatized amino acids, (ii) determination of mass isotopologue distributions of labelled TBDMS-amino acids, and (iii) correction for ^13^C-incorporation concerning the heavy isotope contributions due to the natural abundances in the TBDMS-moiety and the amino acid atoms. The data for listerial amino acids were normalized by subtracting the contributions from host cell protein due to minor contamination of the bacterial pellet with host cell membrane fragments.

## Supporting Information

Figure S1 **Reconstructed metabolic fluxes** **based on the ^13^C-labelled amino acid isotopologues deriving from [U-^13^C_6_]glucose. Flux 1** describes the formation of [^13^C_3_]-Ala, [^13^C_2_]-Asp and [^13^C_2_]-Glu isotopologues by glycolysis and the canonical TCA cycle using unlabeled OAA (deriving from glutamine) and [^13^C_2_]-Ac-CoA from [U-^13^C_6_]-glucose. **Flux 2** describes the formation of the [^13^C_1_]-, [^13^C_2_]- and [^13^C_3_]-Glu isotopologues and of the [^13^C_1_]-, [^13^C_3_]-, and [^13^C_4_]-Asp isotopologues via glycolysis, PYC-derived ^13^C-labeled OAA intermediates and unlabeled Ac-CoA (from glutamine-derived pyruvate (Pyr). The intermediates deriving from citrate are converted to the corresponding ^13^C-Asp and ^13^C_3_-Glu isotopologues by ACL and ICD-1 and ICD-2, respectively. Intermediates predominantly deriving from ^13^C-glucose are written in red letters, while those predominantly deriving from unlabeled glutamine are in black letters and those deriving from ^13^C-glucose and unlabeled glutamine in green letters. The red dots indicate the number of ^13^C-atoms in the respective compound.
(TIF)

Figure S2 **Reconstructed metabolic fluxes based on the ^13^C-labelled amino acid isotopologues deriving from [U-^13^C_5_]glutamine. Flux 1** shows the formation of [^13^C_5_]-, [^13^C_3_]-Glu and [^13^C_4_]-Asp via glutaminolysis, TCA cycle reactions, and ACL-catalyzed reaction to [^13^C_4_]-OAA. Intermediates deriving predominantly from [U-^13^C_5_]glutamine are written in blue letters, while those deriving predominantly from unlabelled glucose are in black letters and those deriving from [U-^13^C_5_]glutamine and unlabelled glucose in green letters. **Flux 2** shows the formation of [^13^C_5_]-Glu and [^13^C_3_]-Asp isotopologues via glutaminolysis, citrate production by isocitrate dehydrogenase (ICD2)-dependent carboxylation of α-KG and citrate conversion to α-KG and OAA by cytosolic ACL and ICD1. Intermediates deriving predominantly from [U-^13^C_5_]glutamine are written in blue letters, while CO_2_ as well as the intermediates deriving predominantly from catabolism of unlabelled glucose are in black letters and those deriving from [U-^13^C_5_]glutamine and unlabelled CO_2_ are in green letters. **Flux 3** describes the formation of [^13^C_2_]-Glu, [^13^C_2_]- and [^13^C_4_]-Asp isotopologues via glutaminolysis, canonical TCA cycle reactions to [^13^C_4_]-malate, conversion to [^13^C_3_]-pyruvate and ^13^CO_2_ generation of [^13^C_2_]-Ac-CoA by PDH, condensation to unlabelled OAA (from unlabelled glucose) yielding [^13^C_2_]-citrate which is further converted by ACL to [^13^C_2_]-OAA and by ICD1 to [^13^C_2_]-α-KG. **Flux 4** describes the formation of [^13^C_1_]-Glu and [^13^C_1_]-Asp isotopomers via glutaminolysis, canonical TCA reactions yielding (among other intermediates) ^13^CO_2_ which is used for PYC-catalyzed carboxylation of unlabelled pyruvate to [^13^C_1_]-OAA, giving rise together with unlabelled Ac-CoA (from unlabelled Glc) to [^13^C_1_]-citrate and further to [^13^C_1_]-OAA and [^13^C_1_]-α-KG. The blue dots indicate the number of ^13^C-atoms in the respective compound. For further details and abbreviations, see main text.
(TIF)

Table S1 **^13^C-Isotopologue abundance in mol% of protein derived amino acids from experiments with uninfected and** ***Listeria monocytogenes*** **-infected BMM and J774A.1 macrophages with 11 mM [U-^13^C_6_]glucose and with or without IFN-γ.** (I) and (II) represent two biological experiments. Isotopologues are described by an extended binary code: 1 represents a ^13^C-atom, 0 stands for ^12^C, X is unknown. Y is unknown, but for a given number (outside the brackets) it represents a ^13^C-atom. Data from host cells represent mean values of three measurements (with S.D.) of cell lysate. Data from intracellular *Listeria monocytogenes* represent calculated values of the bacterial fraction (spill over factor of host cell pellet was determined as described under Methods); n.d. means not determined.
(PDF)

Table S2 **^13^C-Isotopologue abundance in mol% of protein derived amino acids from experiments with uninfected and** ***Listeria monocytogenes*** **-infected BMM and J774A.1 macrophages with 2 mM [U-^13^C_5_]glutamine and with or without IFN-γ.** (I) and (II) represent two biological experiments. Isotopologues are described by an extended binary code: 1 represents a ^13^C-atom, 0 stands for ^12^C, X is unknown. Y is unknown, but for a given number (outside the brackets) it represents a ^13^C-atom. Data from host cells represent mean values of three measurements (with S.D.) of cell lysate. Data from intracellular *Listeria monocytogenes* represent calculated values of the bacterial fraction (spill-over factor of host cell pellet was determined as described under Methods); n.d. means not determined.
(PDF)
