# Supplementary figures and images for: Metabolic Responses of Primary and Transformed Cells to Intracellular Listeria monocytogenes
Source: PLoS One. 2012 Dec 21;7(12):e52378. doi: 10.1371/journal.pone.0052378 (PMC3528701; doi:10.1371/journal.pone.0052378)

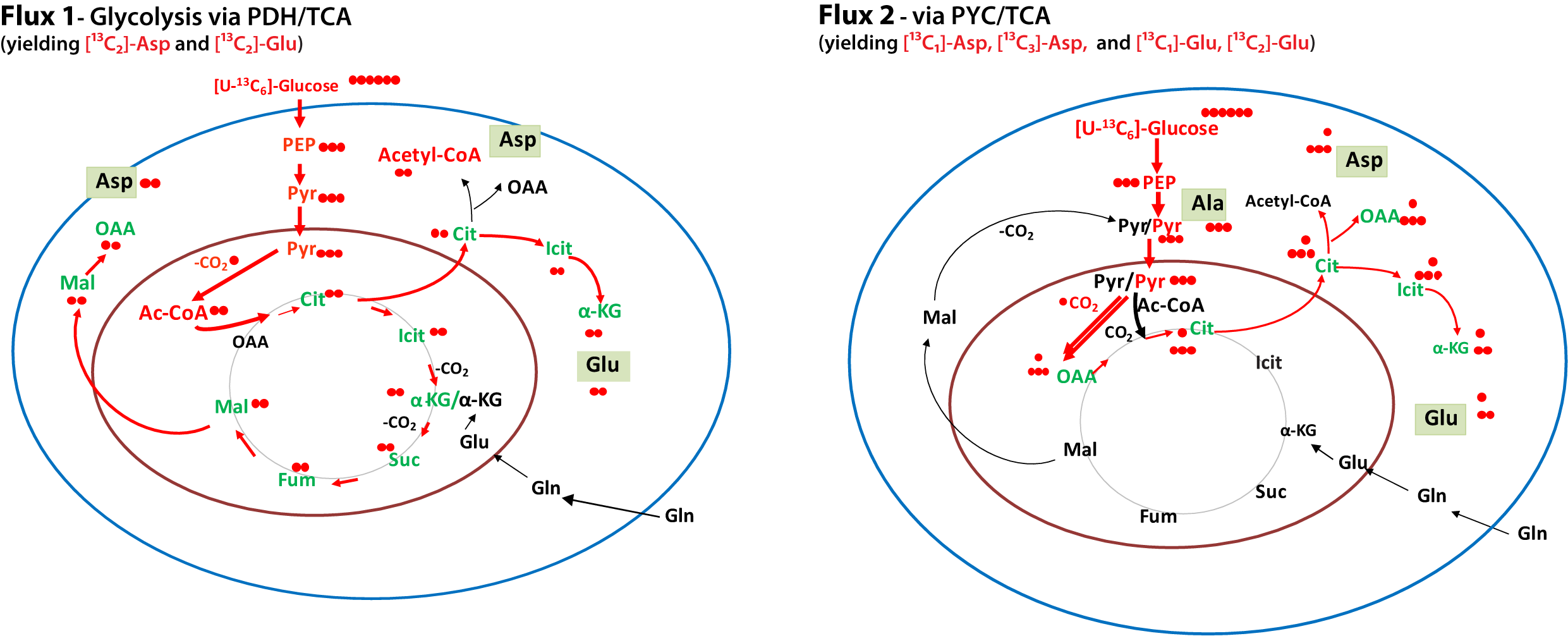

Supplement: Figure S1 — Reconstructed metabolic fluxes based on the 13C-labelled amino acid isotopologues deriving from [U-13C6]glucose. Flux 1 describes the formation of [13C3]-Ala, [13C2]-Asp and [13C2]-Glu isotopologues by glycolysis and the canonical TCA cycle using unlabeled OAA (deriving from glutamine) and [13C2]-Ac-CoA from [U-13C6]-glucose. Flux 2 describes the formation of the [13C1]-, [13C2]- and [13C3]-Glu isotopologues and of the [13C1]-, [13C3]-, and [13C4]-Asp isotopologues via glycolysis, PYC-derived 13C-labeled OAA intermediates and unlabeled Ac-CoA (from glutamine-derived pyruvate (Pyr). The intermediates deriving from citrate are converted to the corresponding 13C-Asp and 13C3-Glu isotopologues by ACL and ICD-1 and ICD-2, respectively. Intermediates predominantly deriving from 13C-glucose are written in red letters, while those predominantly deriving from unlabeled glutamine are in black letters and those deriving from 13C-glucose and unlabeled glutamine in green letters. The red dots indicate the number of 13C-atoms in the respective compound. (TIF) [file pone.0052378.s001.tif]

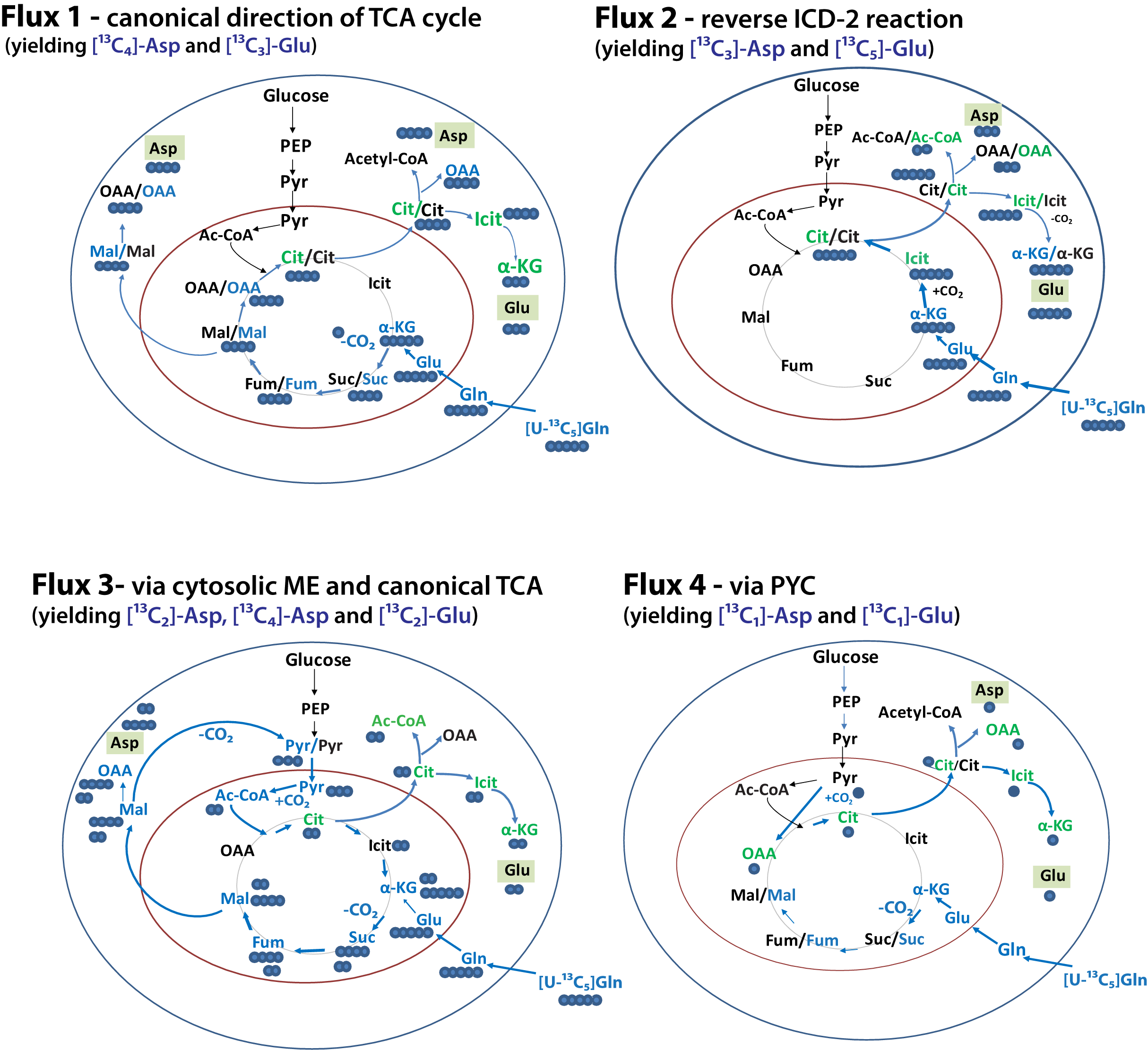

Supplement: Figure S2 — Reconstructed metabolic fluxes based on the 13C-labelled amino acid isotopologues deriving from [U-13C5]glutamine. Flux 1 shows the formation of [13C5]-, [13C3]-Glu and [13C4]-Asp via glutaminolysis, TCA cycle reactions, and ACL-catalyzed reaction to [13C4]-OAA. Intermediates deriving predominantly from [U-13C5]glutamine are written in blue letters, while those deriving predominantly from unlabelled glucose are in black letters and those deriving from [U-13C5]glutamine and unlabelled glucose in green letters. Flux 2 shows the formation of [13C5]-Glu and [13C3]-Asp isotopologues via glutaminolysis, citrate production by isocitrate dehydrogenase (ICD2)-dependent carboxylation of α-KG and citrate conversion to α-KG and OAA by cytosolic ACL and ICD1. Intermediates deriving predominantly from [U-13C5]glutamine are written in blue letters, while CO2 as well as the intermediates deriving predominantly from catabolism of unlabelled glucose are in black letters and those deriving from [U-13C5]glutamine and unlabelled CO2 are in green letters. Flux 3 describes the formation of [13C2]-Glu, [13C2]- and [13C4]-Asp isotopologues via glutaminolysis, canonical TCA cycle reactions to [13C4]-malate, conversion to [13C3]-pyruvate and 13CO2 generation of [13C2]-Ac-CoA by PDH, condensation to unlabelled OAA (from unlabelled glucose) yielding [13C2]-citrate which is further converted by ACL to [13C2]-OAA and by ICD1 to [13C2]-α-KG. Flux 4 describes the formation of [13C1]-Glu and [13C1]-Asp isotopomers via glutaminolysis, canonical TCA reactions yielding (among other intermediates) 13CO2 which is used for PYC-catalyzed carboxylation of unlabelled pyruvate to [13C1]-OAA, giving rise together with unlabelled Ac-CoA (from unlabelled Glc) to [13C1]-citrate and further to [13C1]-OAA and [13C1]-α-KG. The blue dots indicate the number of 13C-atoms in the respective compound. For further details and abbreviations, see main text. (TIF) [file pone.0052378.s002.tif]
