# Supplementary material for: Metabolic Responses of Primary and Transformed Cells to Intracellular Listeria monocytogenes
Source: PLoS One. 2012 Dec 21;7(12):e52378. doi: 10.1371/journal.pone.0052378 (PMC3528701; doi:10.1371/journal.pone.0052378)
Supplement: Table S1 — 13C-Isotopologue abundance in mol% of protein derived amino acids from experiments with uninfected and Listeria monocytogenes -infected BMM and J774A.1 macrophages with 11 mM [U-13C6]glucose and with or without IFN-γ. (I) and (II) represent two biological experiments. Isotopologues are described by an extended binary code: 1 represents a 13C-atom, 0 stands for 12C, X is unknown. Y is unknown, but for a given number (outside the brackets) it represents a 13C-atom. Data from host cells represent mean values of three measurements (with S.D.) of cell lysate. Data from intracellular Listeria monocytogenes represent calculated values of the bacterial fraction (spill over factor of host cell pellet was determined as described under Methods); n.d. means not determined. (PDF) [file pone.0052378.s003.pdf]

Table S1. ^{13}C-Isotopologue abundance in mol% of protein derived amino acids from experiments with uninfected and *Listeria monocytogenes*-infected BMM and J774A.1 macrophages with 11 mM **[U-$^{13}C_6$]glucose** and with or without IFN-γ. (I) and (II) represent two independent biological experiments. Isotopologues are described by an extended binary code: 1 represents a ^{13}C-atom, 0 stands for ^{12}C, X is unknown and Y is unknown, but for a given number (outside the brackets) Y represents a ^{13}C-atom. Data from host cells represent mean values of three measurements (with S.D.). n.d. means not determined.

		11 mM [U-$^{13}C_6$]glucose (-IFN-γ / uninfected) BMM (I)			BMM (II)		
Ala-260	{000}	99,69 %	±	0,01 %	99,53 %	±	0,03 %
	{YYY}1	0,01 %	±	0,02 %	0,07 %	±	0,04 %
	{YYY}2	0,01 %	±	0,01 %	0,04 %	±	0,02 %
	{111}	0,30 %	±	0,02 %	0,37 %	±	0,01 %
Asp-418	{0000}	99,88 %	±	0,13 %	98,92 %	±	0,26 %
	{YYYY}1	0,07 %	±	0,12 %	0,92 %	±	0,24 %
	{YYYY}2	0,00 %	±	0,00 %	0,07 %	±	0,07 %
	{YYYY}3	0,05 %	±	0,02 %	0,09 %	±	0,05 %
	{1111}	0,00 %	±	0,00 %	0,00 %	±	0,00 %
Glu-432	{00000}	99,32 %	±	0,07 %	97,99 %	±	0,32 %
	{YYYYY}1	0,00 %	±	0,00 %	0,91 %	±	0,09 %
	{YYYYY}2	0,62 %	±	0,08 %	1,07 %	±	0,28 %
	{YYYYY}3	0,03 %	±	0,01 %	0,01 %	±	0,02 %
	{YYYYY}4	0,02 %	±	0,03 %	0,00 %	±	0,00 %
	{11111}	0,01 %	±	0,02 %	0,02 %	±	0,00 %
Gly-246	{00}	100,00 %	±	0,00 %	99,74 %	±	0,04 %
	{YY}1	0,00 %	±	0,00 %	0,14 %	±	0,03 %
	{11}	0,00 %	±	0,00 %	0,12 %	±	0,02 %
Ser-390	{000}	99,16 %	±	0,69 %	99,85 %	±	0,00 %
	{YYY}1	0,75 %	±	0,72 %	0,00 %	±	0,00 %
	{YYY}2	0,04 %	±	0,04 %	0,00 %	±	0,00 %
	{111}	0,05 %	±	0,08 %	0,15 %	±	0,00 %

		11 mM [U-$^{13}C_6$]glucose					
		(+IFN-γ / uninfected)					
		BMM (I)			BMM (II)		
Ala-260	{000}	99,46 %	±	0,07 %	98,69 %	±	0,07 %
	{YYY}1	0,00 %	±	0,00 %	0,17 %	±	0,07 %
	{YYY}2	0,07 %	±	0,05 %	0,13 %	±	0,02 %
	{111}	0,46 %	±	0,02 %	1,01 %	±	0,01 %
Asp-418	{0000}	99,86 %	±	0,05 %	98,16 %	±	0,11 %
	{YYYY}1	0,05 %	±	0,09 %	1,27 %	±	0,10 %
	{YYYY}2	0,00 %	±	0,00 %	0,40 %	±	0,05 %
	{YYYY}3	0,08 %	±	0,05 %	0,17 %	±	0,01 %
	{1111}	0,00 %	±	0,00 %	0,00 %	±	0,00 %
Glu-432	{00000}	99,17 %	±	0,09 %	96,64 %	±	0,16 %
	{YYYYY}1	0,00 %	±	0,00 %	1,36 %	±	0,15 %
	{YYYYY}2	0,73 %	±	0,09 %	1,77 %	±	0,06 %
	{YYYYY}3	0,07 %	±	0,04 %	0,12 %	±	0,03 %
	{YYYYY}4	0,00 %	±	0,00 %	0,05 %	±	0,01 %
	{11111}	0,02 %	±	0,00 %	0,06 %	±	0,00 %
Gly-246	{00}	99,99 %	±	0,03 %	99,44 %	±	0,03 %
	{YY}1	0,00 %	±	0,00 %	0,23 %	±	0,01 %
	{11}	0,01 %	±	0,03 %	0,33 %	±	0,02 %
Ser-390	{000}	99,41 %	±	0,17 %	99,52 %	±	0,06 %
	{YYY}1	0,49 %	±	0,15 %	0,05 %	±	0,08 %
	{YYY}2	0,00 %	±	0,00 %	0,00 %	±	0,00 %
	{111}	0,10 %	±	0,03 %	0,43 %	±	0,04 %

11 mM [U-$^{13}C_6$]glucose

(-IFN-γ / infected)

Fragment	Isotopomer	**BMM (I)**	***L.m.* (I)**	**BMM (II)**	***L.m.* (II)**
Ala-260	{000}	99,02 % ± 0,10 %	93,23 %	97,69 % ± 0,05 %	90,00 %
	{YYY}1	0,06 % ± 0,11 %	0,00 %	0,45 % ± 0,03 %	0,00 %
	{YYY}2	0,03 % ± 0,05 %	0,28 %	0,24 % ± 0,03 %	0,42 %
	{111}	0,88 % ± 0,02 %	6,49 %	1,61 % ± 0,00 %	9,57 %
Asp-418	{0000}	99,30 % ± 0,10 %	93,68 %	96,77 % ± 0,10 %	88,79 %
	{YYYY}1	0,13 % ± 0,12 %	0,13 %	1,47 % ± 0,07 %	0,00 %
	{YYYY}2	0,09 % ± 0,08 %	0,42 %	1,04 % ± 0,03 %	0,25 %
	{YYYY}3	0,46 % ± 0,10 %	5,71 %	0,64 % ± 0,01 %	10,93 %
	{1111}	0,02 % ± 0,02 %	0,06 %	0,08 % ± 0,00 %	0,02 %
Glu-432	{00000}	97,52 % ± 0,03 %	96,66 %	92,90 % ± 0,16 %	96,33 %
	{YYYYY}1	0,00 % ± 0,00 %	0,00 %	1,67 % ± 0,11 %	0,00 %
	{YYYYY}2	1,69 % ± 0,06 %	1,04 %	3,88 % ± 0,08 %	0,31 %
	{YYYYY}3	0,40 % ± 0,03 %	0,16 %	0,66 % ± 0,03 %	0,04 %
	{YYYYY}4	0,27 % ± 0,01 %	2,11 %	0,63 % ± 0,01 %	3,32 %
	{11111}	0,12 % ± 0,02 %	0,03 %	0,25 % ± 0,01 %	0,00 %
Gly-246	{00}	100,00 % ± 0,01 %	99,54 %	99,17 % ± 0,17 %	99,27 %
	{YY}1	0,00 % ± 0,00 %	0,00 %	0,39 % ± 0,10 %	0,00 %
	{11}	0,00 % ± 0,01 %	0,46 %	0,44 % ± 0,07 %	0,73 %
His-440	{000000}	n.d.	99,83 %	n.d.	99,65 %
	{YYYYYY}1		0,00 %		0,04 %
	{YYYYYY}2		0,04 %		0,00 %
	{YYYYYY}3		0,02 %		0,03 %
	{YYYYYY}4		0,02 %		0,10 %
	{YYYYYY}5		0,06 %		0,10 %
	{111111}		0,03 %		0,08 %
Ile-200	{X00000}	n.d.	99,11 %	n.d.	99,78 %
	{XYYYYY}1		0,00 %		0,01 %
	{XYYYYY}2		0,35 %		0,00 %
	{XYYYYY}3		0,00 %		0,00 %
	{XYYYYY}4		0,00 %		0,00 %
	{YYYYYY}5		0,53 %		0,00 %
Ile 233	{111111}		0,00 %		0,21 %
Leu-274	{X00000}	n.d.	99,97 %	n.d.	96,30 %
	{XYYYYY}1		0,00 %		0,72 %
	{XYYYYY}2		0,00 %		0,38 %
	{XYYYYY}3		0,01 %		2,23 %
	{XYYYYY}4		0,01 %		0,09 %
	{YYYYYY}5		0,01 %		0,28 %
Leu-233	{111111}		0,00 %		0,00 %
Lys-431	{000000}	n.d.	99,24 %	n.d.	98,82 %
	{YYYYYY}1		0,00 %		0,00 %
	{YYYYYY}2		0,00 %		0,00 %
	{YYYYYY}3		0,45 %		0,59 %
	{YYYYYY}4		0,00 %		0,00 %
	{YYYYYY}5		0,32 %		0,59 %
	{111111}		0,00 %		0,00 %

	11 mM [U-$^{13}C_6$]glucose			
	(-IFN-γ / infected)			
	**BMM (I)**	***L.m.* (I)**	**BMM (II)**	***L.m.* (II)**
Phe-336 000000}	n.d.	99,14 %	n.d.	99,18 %
{YYYYYYYYY}1		0,00 %		0,18 %
{YYYYYYYYY}2		0,08 %		0,00 %
{YYYYYYYYY}3		0,01 %		0,01 %
{YYYYYYYYY}4		0,15 %		0,13 %
{YYYYYYYYY}5		0,03 %		0,08 %
{YYYYYYYYY}6		0,57 %		0,43 %
{YYYYYYYYY}7		0,03 %		0,00 %
{YYYYYYYYY}8		0,00 %		0,00 %
{111111111}		0,00 %		0,00 %
Pro-286 {00000}	n.d.	99,93 %	n.d.	99,50 %
{YYYYY}1		0,01 %		0,00 %
{YYYYY}2		0,00 %		0,00 %
{YYYYY}3		0,04 %		0,02 %
{YYYYY}4		0,00 %		0,48 %
{11111}		0,01 %		0,00 %
Ser-390 {000}	99,14 % ± 0,23 %	98,69 %	99,54 % ± 0,35 %	96,27 %
{YYY}1	0,75 % ± 0,23 %	0,00 %	0,26 % ± 0,37 %	0,87 %
{YYY}2	0,02 % ± 0,02 %	0,00 %	0,00 % ± 0,00 %	0,39 %
{111}	0,08 % ± 0,03 %	1,31 %	0,19 % ± 0,02 %	2,47 %
Thr-404 {0000}	n.d.	95,91 %	n.d.	92,82 %
{YYYY}1		0,73 %		1,35 %
{YYYY}2		0,98 %		0,94 %
{YYYY}3		2,38 %		4,90 %
{1111}		0,00 %		0,00 %
Tyr-466 000000}	n.d.	98,44 %	n.d.	97,43 %
{YYYYYYYYY}1		0,45 %		0,03 %
{YYYYYYYYY}2		0,05 %		0,19 %
{YYYYYYYYY}3		0,04 %		0,04 %
{YYYYYYYYY}4		0,00 %		0,01 %
{YYYYYYYYY}5		0,12 %		0,29 %
{YYYYYYYYY}6		0,11 %		0,31 %
{YYYYYYYYY}7		0,00 %		0,10 %
{YYYYYYYYY}8		0,10 %		0,20 %
{111111111}		0,70 %		1,39 %
Val-288 {00000}	n.d.	99,82 %	n.d.	99,49 %
{YYYYY}1		0,00 %		0,19 %
{YYYYY}2		0,05 %		0,06 %
{YYYYY}3		0,03 %		0,04 %
{YYYYY}4		0,03 %		0,02 %
{11111}		0,08 %		0,20 %

		11 mM [U-$^{13}C_6$]glucose			
		(+IFN-γ / infected)			
		BMM (I)	*L.m.* (I)	BMM (II)	*L.m.* (II)
Ala-260	{000}	98,37 % ± 0,05 %	94,64 %	97,15 % ± 0,16 %	91,64 %
	{YYY}1	0,01 % ± 0,02 %	0,00 %	0,35 % ± 0,15 %	0,00 %
	{YYY}2	0,03 % ± 0,01 %	0,23 %	0,22 % ± 0,01 %	0,32 %
	{111}	1,59 % ± 0,03 %	5,13 %	2,28 % ± 0,01 %	8,04 %
Asp-418	{0000}	98,88 % ± 0,20 %	95,39 %	96,88 % ± 0,02 %	90,12 %
	{YYYY}1	0,22 % ± 0,18 %	0,19 %	1,32 % ± 0,03 %	0,00 %
	{YYYY}2	0,31 % ± 0,07 %	0,29 %	1,10 % ± 0,03 %	0,00 %
	{YYYY}3	0,54 % ± 0,05 %	4,13 %	0,63 % ± 0,01 %	9,89 %
	{1111}	0,05 % ± 0,01 %	0,01 %	0,08 % ± 0,01 %	0,00 %
Glu-432	{00000}	96,73 % ± 0,11 %	97,63 %	93,33 % ± 0,06 %	96,63 %
	{YYYYY}1	0,14 % ± 0,06 %	0,00 %	1,58 % ± 0,07 %	0,00 %
	{YYYYY}2	2,07 % ± 0,23 %	0,78 %	3,59 % ± 0,06 %	0,27 %
	{YYYYY}3	0,55 % ± 0,10 %	0,13 %	0,66 % ± 0,01 %	0,09 %
	{YYYYY}4	0,37 % ± 0,03 %	1,43 %	0,60 % ± 0,01 %	3,00 %
	{11111}	0,14 % ± 0,01 %	0,03 %	0,23 % ± 0,00 %	0,00 %
Gly-246	{00}	99,90 % ± 0,07 %	99,74 %	99,14 % ± 0,07 %	99,56 %
	{YY}1	0,00 % ± 0,00 %	0,00 %	0,40 % ± 0,07 %	0,00 %
	{11}	0,10 % ± 0,07 %	0,26 %	0,46 % ± 0,01 %	0,44 %
His-440	{000000}	n.d.	98,96 %	n.d.	99,75 %
	{YYYYYY}1		0,00 %		0,00 %
	{YYYYYY}2		0,67 %		0,01 %
	{YYYYYY}3		0,28 %		0,04 %
	{YYYYYY}4		0,02 %		0,10 %
	{YYYYYY}5		0,04 %		0,07 %
	{111111}		0,03 %		0,03 %
Ile-200	{X00000}	n.d.	99,35 %	n.d.	99,48 %
	{XYYYYY}1		0,00 %		0,30 %
	{XYYYYY}2		0,37 %		0,00 %
	{XYYYYY}3		0,02 %		0,00 %
	{XYYYYY}4		0,00 %		0,00 %
	{YYYYYY}5		0,26 %		0,00 %
Ile 233	{111111}		0,00 %		0,22 %
Leu-274	{X00000}	n.d.	99,98 %	n.d.	91,87 %
	{XYYYYY}1		0,00 %		0,07 %
	{XYYYYY}2		0,00 %		1,55 %
	{XYYYYY}3		0,01 %		5,85 %
	{XYYYYY}4		0,00 %		0,20 %
	{YYYYYY}5		0,01 %		0,45 %
Leu-233	{111111}		0,00 %		0,02 %
Lys-431	{000000}	n.d.	99,50 %	n.d.	99,12 %
	{YYYYYY}1		0,00 %		0,00 %
	{YYYYYY}2		0,10 %		0,00 %
	{YYYYYY}3		0,37 %		0,48 %
	{YYYYYY}4		0,00 %		0,00 %
	{YYYYYY}5		0,03 %		0,40 %
	{111111}		0,00 %		0,00 %

		11 mM [U-$^{13}C_6$]glucose			
		(+IFN-γ / infected)			
		**BMM (I)**	***L.m.* (I)**	**BMM (II)**	***L.m.* (II)**
Phe-336	[illegible]00000000}	n.d.	99,26 %	n.d.	98,93 %
	{YYYYYYYYY}1		0,00 %		0,00 %
	{YYYYYYYYY}2		0,02 %		0,00 %
	{YYYYYYYYY}3		0,00 %		0,00 %
	{YYYYYYYYY}4		0,17 %		0,15 %
	{YYYYYYYYY}5		0,03 %		0,30 %
	{YYYYYYYYY}6		0,52 %		0,53 %
	{YYYYYYYYY}7		0,00 %		0,09 %
	{YYYYYYYYY}8		0,00 %		0,00 %
	{111111111}		0,00 %		0,00 %
Pro-286	{00000}	n.d.	99,67 %	n.d.	99,55 %
	{YYYYY}1		0,00 %		0,00 %
	{YYYYY}2		0,00 %		0,00 %
	{YYYYY}3		0,14 %		0,11 %
	{YYYYY}4		0,20 %		0,34 %
	{11111}		0,00 %		0,00 %
Ser-390	{000}	99,06 % ± 0,04 %	98,17 %	99,43 % ± 0,28 %	97,92 %
	{YYY}1	0,59 % ± 0,10 %	1,02 %	0,25 % ± 0,29 %	0,10 %
	{YYY}2	0,05 % ± 0,09 %	0,05 %	0,00 % ± 0,00 %	0,23 %
	{111}	0,29 % ± 0,09 %	0,76 %	0,31 % ± 0,02 %	1,75 %
Thr-404	{0000}	n.d.	97,25 %	n.d.	93,93 %
	{YYYY}1		0,65 %		1,49 %
	{YYYY}2		1,36 %		0,74 %
	{YYYY}3		0,74 %		3,83 %
	{1111}		0,00 %		0,00 %
Tyr-466	[illegible]00000000}	n.d.	99,01 %	n.d.	99,03 %
	{YYYYYYYYY}1		0,13 %		0,00 %
	{YYYYYYYYY}2		0,02 %		0,18 %
	{YYYYYYYYY}3		0,04 %		0,00 %
	{YYYYYYYYY}4		0,00 %		0,02 %
	{YYYYYYYYY}5		0,15 %		0,25 %
	{YYYYYYYYY}6		0,10 %		0,26 %
	{YYYYYYYYY}7		0,00 %		0,15 %
	{YYYYYYYYY}8		0,12 %		0,12 %
	{111111111}		0,44 %		1,22 %
Val-288	{00000}	n.d.	99,90 %	n.d.	99,68 %
	{YYYYY}1		0,00 %		0,27 %
	{YYYYY}2		0,00 %		0,02 %
	{YYYYY}3		0,01 %		0,00 %
	{YYYYY}4		0,02 %		0,03 %
	{11111}		0,06 %		0,19 %

		11 mM [U-$^{13}C_6$]glucose (-IFN-γ / uninfected) J774A.1 (I)			J774A.1 (II)		
Ala-260	{000}	93,94 %	±	0,11 %	91,01 %	±	0,06 %
	{YYY}1	0,23 %	±	0,10 %	0,04 %	±	0,04 %
	{YYY}2	0,27 %	±	0,01 %	0,36 %	±	0,02 %
	{111}	5,56 %	±	0,01 %	8,59 %	±	0,04 %
Asp-418	{0000}	94,80 %	±	0,11 %	93,46 %	±	0,18 %
	{YYYY}1	1,32 %	±	0,09 %	1,04 %	±	0,18 %
	{YYYY}2	2,55 %	±	0,07 %	3,34 %	±	0,07 %
	{YYYY}3	1,17 %	±	0,04 %	1,81 %	±	0,08 %
	{1111}	0,16 %	±	0,00 %	0,36 %	±	0,01 %
Glu-432	{00000}	91,95 %	±	0,19 %	90,85 %	±	0,21 %
	{YYYYY}1	1,10 %	±	0,15 %	0,50 %	±	0,16 %
	{YYYYY}2	5,20 %	±	0,07 %	5,99 %	±	0,04 %
	{YYYYY}3	0,77 %	±	0,02 %	1,21 %	±	0,03 %
	{YYYYY}4	0,73 %	±	0,01 %	1,08 %	±	0,02 %
	{11111}	0,25 %	±	0,01 %	0,37 %	±	0,01 %
Gly-246	{00}	99,45 %	±	0,04 %	99,65 %	±	0,00 %
	{YY}1	0,28 %	±	0,04 %	0,17 %	±	0,03 %
	{11}	0,27 %	±	0,02 %	0,18 %	±	0,03 %
Ser-390	{000}	99,89 %	±	0,01 %	99,74 %	±	0,25 %
	{YYY}1	0,00 %	±	0,00 %	0,17 %	±	0,30 %
	{YYY}2	0,00 %	±	0,00 %	0,00 %	±	0,00 %
	{111}	0,11 %	±	0,01 %	0,09 %	±	0,05 %

		11 mM [U-$^{13}C_6$]glucose	
		(+IFN-γ / uninfected)<br>J774A.1 (I)	J774A.1 (II)
Ala-260	{000}	94,60 % ± 0,08 %	92,06 % ± 0,09 %
	{YYY}1	0,00 % ± 0,00 %	0,00 % ± 0,00 %
	{YYY}2	0,17 % ± 0,04 %	0,30 % ± 0,04 %
	{111}	5,24 % ± 0,06 %	7,64 % ± 0,06 %
Asp-418	{0000}	97,94 % ± 0,05 %	95,80 % ± 0,22 %
	{YYYY}1	0,14 % ± 0,12 %	0,59 % ± 0,17 %
	{YYYY}2	1,23 % ± 0,10 %	2,40 % ± 0,07 %
	{YYYY}3	0,67 % ± 0,02 %	1,05 % ± 0,02 %
	{1111}	0,01 % ± 0,01 %	0,15 % ± 0,00 %
Glu-432	{00000}	97,13 % ± 0,08 %	93,97 % ± 0,15 %
	{YYYYY}1	0,00 % ± 0,00 %	0,16 % ± 0,13 %
	{YYYYY}2	2,31 % ± 0,11 %	4,35 % ± 0,08 %
	{YYYYY}3	0,29 % ± 0,03 %	0,72 % ± 0,05 %
	{YYYYY}4	0,20 % ± 0,03 %	0,61 % ± 0,01 %
	{11111}	0,07 % ± 0,01 %	0,19 % ± 0,00 %
Gly-246	{00}	99,91 % ± 0,01 %	99,60 % ± 0,06 %
	{YY}1	0,06 % ± 0,01 %	0,12 % ± 0,05 %
	{11}	0,03 % ± 0,02 %	0,28 % ± 0,02 %
Ser-390	{000}	99,97 % ± 0,03 %	99,50 % ± 0,16 %
	{YYY}1	0,00 % ± 0,00 %	0,39 % ± 0,15 %
	{YYY}2	0,00 % ± 0,00 %	0,00 % ± 0,00 %
	{111}	0,03 % ± 0,03 %	0,10 % ± 0,00 %

		11 mM [U-$^{13}C_6$]glucose			
		(-IFN-γ/ infected)			
		**J774A.1 (I)**	***L.m.* (I)**	**J774A.1(II)**	***L.m.* (II)**
Ala-260	{000}	93,93 % ± 0,07 %	88,90 %	91,09 % ± 0,05 %	89,94 %
	{YYY}1	0,23 % ± 0,04 %	0,00 %	0,00 % ± 0,00 %	0,06 %
	{YYY}2	0,29 % ± 0,01 %	0,33 %	0,36 % ± 0,05 %	0,41 %
	{111}	5,55 % ± 0,05 %	10,77 %	8,55 % ± 0,01 %	9,59 %
Asp-418	{0000}	96,77 % ± 0,08 %	85,89 %	95,89 % ± 0,27 %	88,19 %
	{YYYY}1	0,80 % ± 0,08 %	0,00 %	0,43 % ± 0,30 %	0,11 %
	{YYYY}2	1,55 % ± 0,03 %	0,00 %	2,20 % ± 0,09 %	0,00 %
	{YYYY}3	0,81 % ± 0,03 %	14,11 %	1,29 % ± 0,02 %	11,68 %
	{1111}	0,07 % ± 0,00 %	0,00 %	0,18 % ± 0,01 %	0,02 %
Glu-432	{00000}	94,99 % ± 0,19 %	95,36%	93,98 % ± 0,11 %	96,69 %
	{YYYYY}1	0,83 % ± 0,18 %	0,00 %	0,00 % ± 0,00 %	0,00 %
	{YYYYY}2	3,35 % ± 0,07 %	0,71 %	4,46 % ± 0,16 %	0,32 %
	{YYYYY}3	0,28 % ± 0,04 %	0,12 %	0,65 % ± 0,05 %	0,02 %
	{YYYYY}4	0,42 % ± 0,01 %	3,81 %	0,72 % ± 0,00 %	2,97 %
	{11111}	0,14 % ± 0,00 %	0,00 %	0,19 % ± 0,00 %	0,00 %
Gly-246	{00}	99,31 % ± 0,05 %	99,24 %	99,55 % ± 0,12 %	99,34 %
	{YY}1	0,23 % ± 0,07 %	0,00 %	0,21 % ± 0,07 %	0,00 %
	{11}	0,46 % ± 0,03 %	0,76 %	0,24 % ± 0,04 %	0,66 %
His-440	{000000}	n.d.	99,51 %	n.d.	99,23 %
	{YYYYYY}1		0,00 %		0,00 %
	{YYYYYY}2		0,00 %		0,00 %
	{YYYYYY}3		0,23 %		0,22 %
	{YYYYYY}4		0,02 %		0,05 %
	{YYYYYY}5		0,17 %		0,26 %
	{111111}		0,07 %		0,25 %
Ile-200	{X00000}	n.d.	99,28 %	n.d.	99,21 %
	{XYYYYY}1		0,49 %		0,39 %
	{XYYYYY}2		0,00 %		0,17 %
	{XYYYYY}3		0,00 %		0,02 %
	{XYYYYY}4		0,02 %		0,00 %
	{YYYYYY}5		0,16 %		0,16 %
Ile 233	{111111}		0,04 %		0,05 %
Leu-274	{X00000}	n.d.	99,08 %	n.d.	99,76 %
	{XYYYYY}1		0,19 %		0,08 %
	{XYYYYY}2		0,36 %		0,12 %
	{XYYYYY}3		0,37 %		0,04 %
	{XYYYYY}4		0,00 %		0,00 %
	{YYYYYY}5		0,00 %		0,00 %
Leu-233	{111111}		0,00 %		0,00 %
Lys-431	{000000}	n.d.	98,46 %	n.d.	98,69 %
	{YYYYYY}1		0,00 %		0,00 %
	{YYYYYY}2		0,00 %		0,00 %
	{YYYYYY}3		0,52 %		0,42 %
	{YYYYYY}4		0,00 %		0,00 %
	{YYYYYY}5		1,02 %		0,89 %
	{111111}		0,00 %		0,00 %

		11 mM [U-$^{13}C_6$]glucose					
		(-IFN-γ/ infected)					
		**J774A.1 (I)**		***L.m.* (I)**	**J774A.1(II)**		***L.m.* (II)**
Phe-336	{000000000}	n.d.		99,20 %	n.d.		99,75 %
	{YYYYYYYYY}1			0,25 %			0,00 %
	{YYYYYYYYY}2			0,00 %			0,00 %
	{YYYYYYYYY}3			0,00 %			0,00 %
	{YYYYYYYYY}4			0,16 %			0,20 %
	{YYYYYYYYY}5			0,08 %			0,03 %
	{YYYYYYYYY}6			0,31 %			0,02 %
	{YYYYYYYYY}7			0,00 %			0,00 %
	{YYYYYYYYY}8			0,00 %			0,00 %
	{111111111}			0,00 %			0,00 %
Pro-286	{00000}	n.d.		99,76 %	n.d.		99,83 %
	{YYYYY}1			0,01 %			0,00 %
	{YYYYY}2			0,00 %			0,00 %
	{YYYYY}3			0,04 %			0,05 %
	{YYYYY}4			0,20 %			0,12 %
	{11111}			0,00 %			0,00 %
Ser-390	{000}	99,79 % ±	0,06 %	96,15 %	99,67 % ±	0,33 %	96,38 %
	{YYY}1	0,04 % ±	0,08 %	0,86 %	0,22 % ±	0,38 %	0,95 %
	{YYY}2	0,00 % ±	0,00 %	0,18 %	0,00 % ±	0,00 %	0,32 %
	{111}	0,17 % ±	0,02 %	2,81 %	0,12 % ±	0,06 %	2,34 %
Thr-404	{0000}	n.d.		91,71 %	n.d.		93,75 %
	{YYYY}1			2,06 %			0,98 %
	{YYYY}2			0,70 %			1,07 %
	{YYYY}3			5,53 %			4,20 %
	{1111}			0,00 %			0,00 %
Tyr-466	{000000000}	n.d.		97,24 %	n.d.		97,62 %
	{YYYYYYYYY}1			0,12 %			0,01 %
	{YYYYYYYYY}2			0,18 %			0,19 %
	{YYYYYYYYY}3			0,01 %			0,03 %
	{YYYYYYYYY}4			0,00 %			0,00 %
	{YYYYYYYYY}5			0,29 %			0,27 %
	{YYYYYYYYY}6			0,22 %			0,29 %
	{YYYYYYYYY}7			0,08 %			0,08 %
	{YYYYYYYYY}8			0,26 %			0,25 %
	{111111111}			1,58 %			1,27 %
Val-288	{00000}	n.d.		99,55 %	n.d.		99,70 %
	{YYYYY}1			0,14 %			0,00 %
	{YYYYY}2			0,01 %			0,01 %
	{YYYYY}3			0,01 %			0,03 %
	{YYYYY}4			0,02 %			0,01 %
	{11111}			0,27 %			0,25 %

		11 mM [U-$^{13}C_6$]glucose			
		(+IFN-γ/ infected)			
		**J774A.1 (I)**	***L.m.* (I)**	**J774A.1 (II)**	***L.m.* (II)**
Ala-260	{000}	96,93 % ± 0,08 %	96,10 %	94,58 % ± 0,01 %	96,09 %
	{YYY}1	0,12 % ± 0,09 %	0,10 %	0,00 % ± 0,00 %	0,01 %
	{YYY}2	0,16 % ± 0,03 %	0,46 %	0,19 % ± 0,03 %	0,28 %
	{111}	2,79 % ± 0,01 %	3,34 %	5,23 % ± 0,03 %	3,63 %
Asp-418	{0000}	98,72 % ± 0,07 %	95,14 %	98,36 % ± 0,07 %	95,68 %
	{YYYY}1	0,66 % ± 0,06 %	0,00 %	0,08 % ± 0,07 %	0,04 %
	{YYYY}2	0,30 % ± 0,02 %	0,00 %	0,93 % ± 0,08 %	0,01 %
	{YYYY}3	0,32 % ± 0,01 %	4,83 %	0,57 % ± 0,02 %	4,27 %
	{1111}	0,00 % ± 0,00 %	0,02 %	0,06 % ± 0,01 %	0,00 %
Glu-432	{00000}	97,39 % ± 0,19 %	98,87 %	96,54 % ± 0,06 %	99,12 %
	{YYYYY}1	0,73 % ± 0,15 %	0,00 %	0,00 % ± 0,00 %	0,00 %
	{YYYYY}2	1,65 % ± 0,04 %	0,00 %	2,63 % ± 0,07 %	0,00 %
	{YYYYY}3	0,05 % ± 0,02 %	0,00 %	0,41 % ± 0,01 %	0,00 %
	{YYYYY}4	0,10 % ± 0,00 %	1,13 %	0,32 % ± 0,00 %	0,88 %
	{11111}	0,07 % ± 0,01 %	0,00 %	0,10 % ± 0,00 %	0,00 %
Gly-246	{00}	99,17 % ± 0,12 %	99,91 %	99,42 % ± 0,04 %	100,00 %
	{YY}1	0,16 % ± 0,06 %	0,09 %	0,17 % ± 0,04 %	0,00 %
	{11}	0,67 % ± 0,06 %	0,00 %	0,41 % ± 0,01 %	0,00 %
His-440	{000000}	n.d.	98,44 %	n.d.	98,75 %
	{YYYYYY}1		0,00 %		0,52 %
	{YYYYYY}2		0,00 %		0,39 %
	{YYYYYY}3		0,00 %		0,00 %
	{YYYYYY}4		0,00 %		0,13 %
	{YYYYYY}5		0,00 %		0,14 %
	{111111}		1,56 %		0,08 %
Ile-200	{X00000}	n.d.	98,86 %	n.d.	99,33 %
	{XYYYYY}1		0,00 %		0,28 %
	{XYYYYY}2		0,00 %		0,11 %
	{XYYYYY}3		0,00 %		0,18 %
	{XYYYYY}4		0,00 %		0,00 %
	{YYYYYY}5		0,06 %		0,00 %
Ile 233	{111111}		1,08 %		0,10 %
Leu-274	{X00000}	n.d.	99,78 %	n.d.	99,85 %
	{XYYYYY}1		0,00 %		0,01 %
	{XYYYYY}2		0,21 %		0,08 %
	{XYYYYY}3		0,00 %		0,06 %
	{XYYYYY}4		0,00 %		0,00 %
	{YYYYYY}5		0,00 %		0,00 %
Leu-233	{111111}		0,00 %		0,00 %
Lys-431	{000000}	n.d.	96,48 %	n.d.	99,85 %
	{YYYYYY}1		0,00 %		0,03 %
	{YYYYYY}2		0,26 %		0,00 %
	{YYYYYY}3		1,54 %		0,13 %
	{YYYYYY}4		0,29 %		0,00 %
	{YYYYYY}5		0,00 %		0,00 %
	{111111}		1,43 %		0,00 %

		11 mM [U-$^{13}C_6$]glucose			
		(+IFN-γ/ infected)			
		**J774A.1 (I)**	***L.m.* (I)**	**J774A.1 (II)**	***L.m.* (II)**
Phe-336	{000000000}	n.d.	96,67 %	n.d.	99,73 %
	{YYYYYYYYY}1		0,01 %		0,00 %
	{YYYYYYYYY}2		0,00 %		0,00 %
	{YYYYYYYYY}3		0,29 %		0,03 %
	{YYYYYYYYY}4		0,33 %		0,03 %
	{YYYYYYYYY}5		0,16 %		0,10 %
	{YYYYYYYYY}6		2,19 %		0,07 %
	{YYYYYYYYY}7		0,35 %		0,03 %
	{YYYYYYYYY}8		0,00 %		0,02 %
	{111111111}		0,00 %		0,00 %
Pro-286	{00000}	n.d.	99,84 %	n.d.	99,88 %
	{YYYYY}1		0,02 %		0,00 %
	{YYYYY}2		0,00 %		0,00 %
	{YYYYY}3		-0,09 %		0,12 %
	{YYYYY}4		0,07 %		0,00 %
	{11111}		0,16 %		0,00 %
Ser-390	{000}	99,81 % ± 0,09 %	99,49 %	99,52 % ± 0,09 %	99,11 %
	{YYY}1	0,06 % ± 0,10 %	0,00 %	0,37 % ± 0,12 %	0,04 %
	{YYY}2	0,00 % ± 0,00 %	0,00 %	0,00 % ± 0,00 %	0,18 %
	{111}	0,13 % ± 0,04 %	0,51 %	0,10 % ± 0,03 %	0,66 %
Thr-404	{0000}	n.d.	97,66 %	n.d.	97,29 %
	{YYYY}1		0,61 %		0,33 %
	{YYYY}2		1,08 %		2,13 %
	{YYYY}3		0,53 %		0,25 %
	{1111}		0,13 %		0,00 %
Tyr-466	{000000000}	n.d.	98,64 %	n.d.	99,76 %
	{YYYYYYYYY}1		0,02 %		0,16 %
	{YYYYYYYYY}2		0,07 %		0,00 %
	{YYYYYYYYY}3		0,14 %		0,00 %
	{YYYYYYYYY}4		0,00 %		0,00 %
	{YYYYYYYYY}5		0,00 %		0,06 %
	{YYYYYYYYY}6		0,25 %		0,00 %
	{YYYYYYYYY}7		0,25 %		0,00 %
	{YYYYYYYYY}8		0,10 %		0,00 %
	{111111111}		0,53 %		0,00 %
Val-288	{00000}	n.d.	99,01 %	n.d.	99,69 %
	{YYYYY}1		0,35 %		0,00 %
	{YYYYY}2		0,04 %		0,02 %
	{YYYYY}3		0,00 %		0,05 %
	{YYYYY}4		0,03 %		0,03 %
	{11111}		0,57 %		0,20 %
