# Supplementary material for: Metabolic Responses of Primary and Transformed Cells to Intracellular Listeria monocytogenes
Source: PLoS One. 2012 Dec 21;7(12):e52378. doi: 10.1371/journal.pone.0052378 (PMC3528701; doi:10.1371/journal.pone.0052378)
Supplement: Table S2 — 13C-Isotopologue abundance in mol% of protein derived amino acids from experiments with uninfected and Listeria monocytogenes -infected BMM and J774A.1 macrophages with 2 mM [U-13C5]glutamine and with or without IFN-γ. (I) and (II) represent two biological experiments. Isotopologues are described by an extended binary code: 1 represents a 13C-atom, 0 stands for 12C, X is unknown. Y is unknown, but for a given number (outside the brackets) it represents a 13C-atom. Data from host cells represent mean values of three measurements (with S.D.) of cell lysate. Data from intracellular Listeria monocytogenes represent calculated values of the bacterial fraction (spill-over factor of host cell pellet was determined as described under Methods); n.d. means not determined. (PDF) [file pone.0052378.s004.pdf]

Table S2. ^{13}C-Isotopologue abundance in mol% of protein derived amino acids from experiments with uninfected and *Listeria monocytogenes*-infected BMM and J774A.1 macrophages with **2 mM [U^{13}C$_5$]glutamine** and with or without IFN-γ. (I) and (II) represent two biological experiments. Isotopologues are described by an extended binary code: 1 represents a ^{13}C-atom, 0 stands for ^{12}C, X is unknown. Y is unknown, but for a given number (outside the brackets) it represents a ^{13}C-atom. Data from host cells represent mean values of three measurements (with S.D.) of cell lysate. Data from intracellular *Listeria monocytogenes* represent calculated values of the bacterial fraction (spill over factor of host cell pellet was determined as described under Methods); n.d. means not determined.

		**2 mM [U-^{13}C$_5$]glutamine**	
		**(-IFN-γ /uninfected)**	
		**BMM (I)**	**BMM (II)**
Ala-260	{000}	99,88 % ± 0,00 %	99,84 % ± 0,03 %
	{YYY}1	0,00 % ± 0,00 %	0,00 % ± 0,00 %
	{YYY}2	0,00 % ± 0,00 %	0,03 % ± 0,03 %
	{111}	0,12 % ± 0,00 %	0,13 % ± 0,01 %
Asp-418	{0000}	95,16 % ± 0,18 %	94,99 % ± 0,13 %
	{YYYY}1	1,09 % ± 0,19 %	0,67 % ± 0,10 %
	{YYYY}2	0,79 % ± 0,05 %	1,04 % ± 0,11 %
	{YYYY}3	0,32 % ± 0,04 %	0,37 % ± 0,01 %
	{1111}	2,65 % ± 0,02 %	2,94 % ± 0,04 %
Glu-432	{00000}	76,93 % ± 0,12 %	77,02 % ± 0,10 %
	{YYYYY}1	1,11 % ± 0,10 %	0,80 % ± 0,17 %
	{YYYYY}2	0,98 % ± 0,06 %	1,04 % ± 0,10 %
	{YYYYY}3	2,64 % ± 0,03 %	3,02 % ± 0,05 %
	{YYYYY}4	0,81 % ± 0,02 %	0,79 % ± 0,01 %
	{11111}	17,53 % ± 0,01 %	17,33 % ± 0,11 %
Gly-246	{00}	99,84 % ± 0,11 %	99,97 % ± 0,03 %
	{YY}1	0,15 % ± 0,09 %	0,01 % ± 0,01 %
	{11}	0,01 % ± 0,01 %	0,03 % ± 0,03 %
Ser-390	{000}	99,74 % ± 0,17 %	99,28 % ± 0,26 %
	{YYY}1	0,18 % ± 0,22 %	0,66 % ± 0,26 %
	{YYY}2	0,00 % ± 0,00 %	0,00 % ± 0,00 %
	{111}	0,08 % ± 0,05 %	0,06 % ± 0,01 %

		2 mM [U-$^{13}C_5$]glutamine	
		(+IFN-γ /uninfected)	
		BMM (I)	BMM (II)
Ala-260	{000}	99,85 % ± 0,02 %	99,84 % ± 0,01 %
	{YYY}1	0,00 % ± 0,00 %	0,00 % ± 0,00 %
	{YYY}2	0,03 % ± 0,01 %	0,05 % ± 0,00 %
	{111}	0,12 % ± 0,01 %	0,11 % ± 0,01 %
Asp-418	{0000}	93,70 % ± 0,12 %	93,96 % ± 0,08 %
	{YYYY}1	1,27 % ± 0,20 %	0,93 % ± 0,08 %
	{YYYY}2	1,22 % ± 0,07 %	1,14 % ± 0,03 %
	{YYYY}3	0,38 % ± 0,01 %	0,39 % ± 0,04 %
	{1111}	3,44 % ± 0,01 %	3,57 % ± 0,03 %
Glu-432	{00000}	76,14 % ± 0,06 %	76,40 % ± 0,24 %
	{YYYYY}1	1,33 % ± 0,07 %	0,78 % ± 0,34 %
	{YYYYY}2	1,10 % ± 0,06 %	0,96 % ± 0,13 %
	{YYYYY}3	2,93 % ± 0,02 %	3,16 % ± 0,00 %
	{YYYYY}4	0,81 % ± 0,01 %	0,80 % ± 0,02 %
	{11111}	17,68 % ± 0,04 %	17,90 % ± 0,04 %
Gly-246	{00}	99,81 % ± 0,01 %	99,85 % ± 0,01 %
	{YY}1	0,17 % ± 0,02 %	0,12 % ± 0,01 %
	{11}	0,02 % ± 0,02 %	0,03 % ± 0,01 %
Ser-390	{000}	99,68 % ± 0,19 %	99,43 % ± 0,39 %
	{YYY}1	0,25 % ± 0,22 %	0,49 % ± 0,43 %
	{YYY}2	0,00 % ± 0,00 %	0,00 % ± 0,00 %
	{111}	0,07 % ± 0,03 %	0,08 % ± 0,04 %

2 mM [U-$^{13}C_5$]glutamine					
		(-IFN-γ / infected)			
		BMM (I)	***L.m.*** **(I)**	BMM (II)	***L.m.*** **(II)**
Ala-260	{000}	99,86 % ± 0,03 %	100,00 %	99,84 % ± 0,03 %	99,97 %
	{YYY}1	0,00 % ± 0,00 %	0,00 %	0,00 % ± 0,00 %	0,03 %
	{YYY}2	0,03 % ± 0,03 %	0,00 %	0,05 % ± 0,05 %	0,00 %
	{111}	0,10 % ± 0,01 %	0,00 %	0,11 % ± 0,02 %	0,00 %
Asp-418	{0000}	95,54 % ± 0,11 %	99,97 %	95,32 % ± 0,08 %	99,54 %
	{YYYY}1	0,92 % ± 0,10 %	0,00 %	0,60 % ± 0,09 %	0,00 %
	{YYYY}2	0,79 % ± 0,04 %	0,00 %	0,97 % ± 0,02 %	0,11 %
	{YYYY}3	0,26 % ± 0,03 %	0,03 %	0,31 % ± 0,01 %	0,12 %
	{1111}	2,50 % ± 0,00 %	0,00 %	2,80 % ± 0,03 %	0,22 %
Glu-432	{00000}	78,27 % ± 0,22 %	80,18 %	77,17 % ± 0,20 %	87,51 %
	{YYYYY}1	1,23 % ± 0,19 %	0,13 %	0,67 % ± 0,17 %	0,00 %
	{YYYYY}2	0,97 % ± 0,04 %	0,00 %	1,00 % ± 0,04 %	0,11 %
	{YYYYY}3	2,64 % ± 0,04 %	0,14 %	2,86 % ± 0,03 %	0,27 %
	{YYYYY}4	0,73 % ± 0,02 %	0,88 %	0,78 % ± 0,05 %	0,55 %
	{11111}	16,16 % ± 0,13 %	18,67 %	17,53 % ± 0,15 %	11,56 %
Gly-246	{00}	99,82 % ± 0,09 %	99,98 %	99,90 % ± 0,06 %	100,00 %
	{YY}1	0,17 % ± 0,09 %	0,00 %	0,07 % ± 0,04 %	0,00 %
	{11}	0,00 % ± 0,01 %	0,02 %	0,02 % ± 0,03 %	0,00 %
His-440	{000000}	n.d.	99,93 %	n.d.	99,22 %
	{YYYYYY}1		0,00 %		0,07 %
	{YYYYYY}2		0,00 %		0,00 %
	{YYYYYY}3		0,02 %		0,24 %
	{YYYYYY}4		0,03 %		0,48 %
	{YYYYYY}5		0,00 %		0,00 %
	{111111}		0,02 %		0,00 %
Ile-200	{X00000}	n.d.	98,90 %	n.d.	99,65 %
	{XYYYYY}1		0,69 %		0,05 %
	{XYYYYY}2		0,10 %		0,24 %
	{XYYYYY}3		0,00 %		0,00 %
	{XYYYYY}4		0,00 %		0,00 %
	{YYYYYY}5		0,31 %		0,00 %
Ile 233	{111111}		0,01 %		0,05 %
Leu-274	{X00000}	n.d.	99,83 %	n.d.	99,88 %
	{XYYYYY}1		0,00 %		0,07 %
	{XYYYYY}2		0,00 %		0,05 %
	{XYYYYY}3		0,00 %		0,00 %
	{XYYYYY}4		0,00 %		0,00 %
	{YYYYYY}5		0,00 %		0,00 %
Leu-233	{111111}		0,17 %		0,00 %
Lys-431	{000000}	n.d.	99,75 %	n.d.	99,68 %
	{YYYYYY}1		0,00 %		0,00 %
	{YYYYYY}2		0,00 %		0,03 %
	{YYYYYY}3		0,25 %		0,29 %
	{YYYYYY}4		0,00 %		0,00 %
	{YYYYYY}5		0,00 %		0,00 %
	{111111}		0,00 %		0,00 %

		2 mM [U-$^{13}C_5$]glutamine			
		(-IFN-γ / infected)			
		BMM (I)	***L.m.*** **(I)**	BMM (II)	***L.m.*** **(II)**
Phe-336	{000000000}	n.d.	99,65 %	n.d.	99,87 %
	{YYYYYYYYY}1		0,00 %		0,00 %
	{YYYYYYYYY}2		0,00 %		0,00 %
	{YYYYYYYYY}3		0,00 %		0,00 %
	{YYYYYYYYY}4		0,13 %		0,13 %
	{YYYYYYYYY}5		0,02 %		0,00 %
	{YYYYYYYYY}6		0,20 %		0,00 %
	{YYYYYYYYY}7		0,00 %		0,00 %
	{YYYYYYYYY}8		0,00 %		0,00 %
	{111111111}		0,00 %		0,00 %
Pro-286	{00000}	n.d.	97,20 %	n.d.	98,34 %
	{YYYYY}1		0,00 %		0,04 %
	{YYYYY}2		0,00 %		0,00 %
	{YYYYY}3		0,18 %		0,11 %
	{YYYYY}4		0,15 %		0,13 %
	{11111}		2,47 %		1,37 %
Ser-390	{000}	99,78 % ± 0,16 %	99,81 %	99,53 % ± 0,32 %	100,00 %
	{YYY}1	0,11 % ± 0,19 %	0,19 %	0,38 % ± 0,33 %	0,00 %
	{YYY}2	0,00 % ± 0,00 %	0,00 %	0,00 % ± 0,00 %	0,00 %
	{111}	0,11 % ± 0,03 %	0,00 %	0,08 % ± 0,03 %	0,00 %
Thr-404	{0000}	n.d.	97,47 %	n.d.	98,82 %
	{YYYY}1		0,24 %		0,46 %
	{YYYY}2		2,29 %		0,71 %
	{YYYY}3		0,00 %		0,00 %
	{1111}		0,00 %		0,00 %
Tyr-466	{000000000}	n.d.	99,79 %	n.d.	99,79 %
	{YYYYYYYYY}1		0,00 %		0,02 %
	{YYYYYYYYY}2		0,13 %		0,02 %
	{YYYYYYYYY}3		0,01 %		0,01 %
	{YYYYYYYYY}4		0,00 %		0,02 %
	{YYYYYYYYY}5		0,07 %		0,07 %
	{YYYYYYYYY}6		0,00 %		0,01 %
	{YYYYYYYYY}7		0,00 %		0,00 %
	{YYYYYYYYY}8		0,00 %		0,03 %
	{111111111}		0,00 %		0,02 %
Val-288	{00000}	n.d.	99,99 %	n.d.	99,98 %
	{YYYYY}1		0,00 %		0,00 %
	{YYYYY}2		0,00 %		0,00 %
	{YYYYY}3		0,01 %		0,01 %
	{YYYYY}4		0,00 %		0,00 %
	{11111}		0,00 %		0,00 %

		2 mM [U-$^{13}C_5$]glutamine			
		(+IFN-γ / infected)			
		**BMM (I)**	***L.m.* (I)**	**BMM (II)**	***L.m.* (II)**
Ala-260	{000}	99,87 % ± 0,01 %	99,91 %	99,85 % ± 0,04 %	99,94 %
	{YYY}1	0,00 % ± 0,01 %	0,00 %	0,00 % ± 0,00 %	0,00 %
	{YYY}2	0,01 % ± 0,01 %	0,03 %	0,03 % ± 0,02 %	0,06 %
	{111}	0,11 % ± 0,01 %	0,06 %	0,12 % ± 0,01 %	0,00 %
Asp-418	{0000}	94,91 % ± 0,18 %	100,00 %	94,35 % ± 0,21 %	99,41 %
	{YYYY}1	0,96 % ± 0,22 %	0,00 %	0,87 % ± 0,24 %	0,26 %
	{YYYY}2	0,92 % ± 0,05 %	0,00 %	1,06 % ± 0,05 %	0,25 %
	{YYYY}3	0,31 % ± 0,01 %	0,00 %	0,34 % ± 0,04 %	0,00 %
	{1111}	2,90 % ± 0,02 %	0,00 %	3,38 % ± 0,04 %	0,09 %
Glu-432	{00000}	77,55 % ± 0,15 %	85,24 %	77,30 % ± 0,24 %	87,71 %
	{YYYYY}1	1,23 % ± 0,07 %	0,00 %	0,78 % ± 0,18 %	0,14 %
	{YYYYY}2	0,90 % ± 0,05 %	0,02 %	0,94 % ± 0,07 %	0,12 %
	{YYYYY}3	2,65 % ± 0,05 %	0,10 %	3,04 % ± 0,06 %	0,16 %
	{YYYYY}4	0,78 % ± 0,02 %	0,69 %	0,78 % ± 0,03 %	0,55 %
	{11111}	16,89 % ± 0,06 %	13,95 %	17,16 % ± 0,08 %	11,31 %
Gly-246	{00}	99,76 % ± 0,05 %	99,98 %	99,89 % ± 0,09 %	99,98 %
	{YY}1	0,21 % ± 0,03 %	0,00 %	0,09 % ± 0,08 %	0,00 %
	{11}	0,03 % ± 0,01 %	0,02 %	0,02 % ± 0,02 %	0,02 %
His-440	{000000}	n.d.	99,58 %	n.d.	n.d.
	{YYYYYY}1		0,00 %		
	{YYYYYY}2		0,00 %		
	{YYYYYY}3		0,11 %		
	{YYYYYY}4		0,28 %		
	{YYYYYY}5		0,03 %		
	{111111}		0,00 %		
Ile-200	{X00000}	n.d.	98,82 %	n.d.	97,22 %
	{XYYYYY}1		0,53 %		0,06 %
	{XYYYYY}2		0,03 %		0,72 %
	{XYYYYY}3		0,00 %		0,32 %
	{XYYYYY}4		0,00 %		0,07 %
	{YYYYYY}5		0,34 %		1,49 %
Ile 233	{111111}		0,29 %		0,12 %
Leu-274	{X00000}	n.d.	99,74 %	n.d.	99,25 %
	{XYYYYY}1		0,05 %		0,00 %
	{XYYYYY}2		0,06 %		0,35 %
	{XYYYYY}3		0,01 %		0,08 %
	{XYYYYY}4		0,00 %		0,15 %
	{YYYYYY}5		0,14 %		0,00 %
Leu-233	{111111}		0,00 %		0,17 %
Lys-431	{000000}	n.d.	99,82 %	n.d.	n.d.
	{YYYYYY}1		0,00 %		
	{YYYYYY}2		0,00 %		
	{YYYYYY}3		0,18 %		
	{YYYYYY}4		0,00 %		
	{YYYYYY}5		0,00 %		
	{111111}		0,00 %		

		2 mM [U-$^{13}C_5$]glutamine			
		(+IFN-γ / infected)			
		BMM (I)	*L.m.* (I)	BMM (II)	*L.m.* (II)
Phe-336	{000000000}	n.d.	99,53 %	n.d.	99,68 %
	{YYYYYYYYY}1		0,00 %		0,00 %
	{YYYYYYYYY}2		0,00 %		0,00 %
	{YYYYYYYYY}3		0,00 %		0,03 %
	{YYYYYYYYY}4		0,18 %		0,10 %
	{YYYYYYYYY}5		0,03 %		0,09 %
	{YYYYYYYYY}6		0,23 %		0,03 %
	{YYYYYYYYY}7		0,02 %		0,04 %
	{YYYYYYYYY}8		0,00 %		0,00 %
	{111111111}		0,00 %		0,03 %
Pro-286	{00000}	n.d.	98,54 %	n.d.	98,96 %
	{YYYYY}1		0,07 %		0,00 %
	{YYYYY}2		0,00 %		0,09 %
	{YYYYY}3		0,10 %		0,01 %
	{YYYYY}4		0,11 %		0,01 %
	{11111}		1,17 %		0,93 %
Ser-390	{000}	99,69 % ± 0,18 %	99,69 %	99,56 % ± 0,35 %	99,89 %
	{YYY}1	0,22 % ± 0,19 %	0,31 %	0,35 % ± 0,37 %	0,05 %
	{YYY}2	0,00 % ± 0,00 %	0,00 %	0,00 % ± 0,00 %	0,00 %
	{111}	0,09 % ± 0,02 %	0,00 %	0,08 % ± 0,02 %	0,06 %
Thr-404	{0000}	n.d.	97,64 %	n.d.	99,00 %
	{YYYY}1		1,26 %		0,30 %
	{YYYY}2		1,10 %		0,70 %
	{YYYY}3		0,00 %		0,00 %
	{1111}		0,00 %		0,00 %
Tyr-466	{000000000}	n.d.	99,75 %	n.d.	98,37 %
	{YYYYYYYYY}1		0,02 %		0,00 %
	{YYYYYYYYY}2		0,15 %		0,82 %
	{YYYYYYYYY}3		0,00 %		0,02 %
	{YYYYYYYYY}4		0,00 %		0,05 %
	{YYYYYYYYY}5		0,06 %		0,15 %
	{YYYYYYYYY}6		0,01 %		0,00 %
	{YYYYYYYYY}7		0,00 %		0,06 %
	{YYYYYYYYY}8		0,00 %		0,46 %
	{111111111}		0,01 %		0,05 %
Val-288	{00000}	n.d.	99,96 %	n.d.	99,91 %
	{YYYYY}1		0,00 %		0,00 %
	{YYYYY}2		0,02 %		0,07 %
	{YYYYY}3		0,01 %		0,00 %
	{YYYYY}4		0,00 %		0,00 %
	{11111}		0,01 %		0,02 %

		2 mM [U-$^{13}C_5$]glutamine			
		(-IFN-γ / uninfected)			
		J774A.1 (I)		J774A.1 (II)	
Ala-260	{000}	99,50 % ±	0,07 %	99,38 % ±	0,06 %
	{YYY}1	0,03 % ±	0,06 %	0,00 % ±	0,00 %
	{YYY}2	0,08 % ±	0,02 %	0,12 % ±	0,06 %
	{111}	0,39 % ±	0,01 %	0,51 % ±	0,00 %
Asp-418	{0000}	80,75 % ±	0,03 %	81,80 % ±	0,09 %
	{YYYY}1	1,34 % ±	0,15 %	0,79 % ±	0,10 %
	{YYYY}2	2,97 % ±	0,10 %	2,58 % ±	0,18 %
	{YYYY}3	2,69 % ±	0,02 %	2,72 % ±	0,03 %
	{1111}	12,25 % ±	0,07 %	12,11 % ±	0,03 %
Glu-432	{00000}	58,35 % ±	0,19 %	60,85 % ±	0,26 %
	{YYYYY}1	0,87 % ±	0,06 %	0,38 % ±	0,08 %
	{YYYYY}2	1,22 % ±	0,05 %	1,13 % ±	0,02 %
	{YYYYY}3	4,57 % ±	0,03 %	3,93 % ±	0,05 %
	{YYYYY}4	1,62 % ±	0,00 %	1,53 % ±	0,02 %
	{11111}	33,37 % ±	0,08 %	32,19 % ±	0,14 %
Gly-246	{00}	99,75 % ±	0,10 %	99,77 % ±	0,08 %
	{YY}1	0,22 % ±	0,08 %	0,20 % ±	0,07 %
	{11}	0,03 % ±	0,03 %	0,03 % ±	0,03 %
Ser-390	{000}	99,84 % ±	0,11 %	99,78 % ±	0,16 %
	{YYY}1	0,05 % ±	0,09 %	0,08 % ±	0,15 %
	{YYY}2	0,00 % ±	0,00 %	0,01 % ±	0,02 %
	{111}	0,10 % ±	0,02 %	0,13 % ±	0,01 %

		2 mM [U-$^{13}C_5$]glutamine			
		(+IFN-γ / uninfected)			
		J774A.1 (I)		J774A.1 (II)	
Ala-260	{000}	99,64 % ±	0,03 %	99,57 % ±	0,05 %
	{YYY}1	0,00 % ±	0,00 %	0,00 % ±	0,00 %
	{YYY}2	0,03 % ±	0,03 %	0,10 % ±	0,08 %
	{111}	0,33 % ±	0,01 %	0,33 % ±	0,03 %
Asp-418	{0000}	87,70 % ±	0,11 %	88,05 % ±	0,17 %
	{YYYY}1	0,28 % ±	0,06 %	0,11 % ±	0,10 %
	{YYYY}2	1,14 % ±	0,04 %	1,06 % ±	0,09 %
	{YYYY}3	2,31 % ±	0,01 %	2,18 % ±	0,04 %
	{1111}	8,58 % ±	0,03 %	8,60 % ±	0,05 %
Glu-432	{00000}	64,08 % ±	0,13 %	66,01 % ±	0,07 %
	{YYYYY}1	0,01 % ±	0,03 %	0,06 % ±	0,09 %
	{YYYYY}2	0,61 % ±	0,04 %	0,55 % ±	0,13 %
	{YYYYY}3	2,34 % ±	0,01 %	2,23 % ±	0,01 %
	{YYYYY}4	1,47 % ±	0,01 %	1,35 % ±	0,04 %
	{11111}	31,48 % ±	0,12 %	29,80 % ±	0,09 %
Gly-246	{00}	99,84 % ±	0,09 %	99,95 % ±	0,05 %
	{YY}1	0,12 % ±	0,06 %	0,04 % ±	0,06 %
	{11}	0,04 % ±	0,04 %	0,01 % ±	0,02 %
Ser-390	{000}	99,89 % ±	0,01 %	99,62 % ±	0,30 %
	{YYY}1	0,00 % ±	0,00 %	0,30 % ±	0,34 %
	{YYY}2	0,00 % ±	0,00 %	0,00 % ±	0,00 %
	{111}	0,11 % ±	0,01 %	0,09 % ±	0,04 %

		2 mM [U-$^{13}C_5$]glutamine			
		(-IFN-γ / infected)			
		J774A.1 (I)	*L.m.* (I)	J774A.1(II)	*L.m.* (II)
Ala-260	{000}	99,61 % ± 0,01 %	100,00 %	99,46 % ± 0,08 %	99,98 %
	{YYY}1	0,01 % ± 0,01 %	0,00 %	0,02 % ± 0,04 %	0,02 %
	{YYY}2	0,05 % ± 0,01 %	0,00 %	0,09 % ± 0,03 %	0,01 %
	{111}	0,33 % ± 0,00 %	0,00 %	0,43 % ± 0,02 %	0,00 %
Asp-418	{0000}	82,20 % ± 0,08 %	99,98 %	84,16 % ± 0,22 %	100,00 %
	{YYYY}1	1,25 % ± 0,08 %	0,02 %	0,81 % ± 0,15 %	0,00 %
	{YYYY}2	2,59 % ± 0,06 %	0,00 %	2,28 % ± 0,06 %	0,00 %
	{YYYY}3	2,47 % ± 0,01 %	0,00 %	1,98 % ± 0,05 %	0,00 %
	{1111}	11,48 % ± 0,05 %	0,00 %	10,78 % ± 0,10 %	0,00 %
Glu-432	{00000}	61,45 % ± 0,25 %	97,95 %	64,93 % ± 0,16 %	95,83 %
	{YYYYY}1	0,71 % ± 0,15 %	0,00 %	0,34 % ± 0,10 %	0,00 %
	{YYYYY}2	1,16 % ± 0,02 %	0,00 %	0,88 % ± 0,12 %	0,09 %
	{YYYYY}3	4,15 % ± 0,01 %	0,00 %	3,57 % ± 0,01 %	0,00 %
	{YYYYY}4	1,48 % ± 0,01 %	0,11 %	1,36 % ± 0,02 %	0,20 %
	{11111}	31,04 % ± 0,09 %	1,94 %	28,91 % ± 0,09 %	3,88 %
Gly-246	{00}	99,74 % ± 0,03 %	100,00 %	99,90 % ± 0,09 %	100,00 %
	{YY}1	0,23 % ± 0,02 %	0,00 %	0,08 % ± 0,07 %	0,00 %
	{11}	0,03 % ± 0,01 %	0,00 %	0,02 % ± 0,02 %	0,00 %
His-440	{000000}	n.d.	99,84 %	n.d.	99,10 %
	{YYYYYY}1		0,00 %		0,00 %
	{YYYYYY}2		0,00 %		0,01 %
	{YYYYYY}3		0,02 %		0,72 %
	{YYYYYY}4		0,03 %		0,06 %
	{YYYYYY}5		0,08 %		0,08 %
	{111111}		0,03 %		0,04 %
Ile-200	{X00000}	n.d.	98,97 %	n.d.	98,82 %
	{XYYYYY}1		0,58 %		0,62 %
	{XYYYYY}2		0,23 %		0,19 %
	{XYYYYY}3		0,00 %		0,00 %
	{XYYYYY}4		0,00 %		0,01 %
	{YYYYYY}5		0,00 %		0,16 %
Ile 233	{111111}		0,21 %		0,20 %
Leu-274	{X00000}	n.d.	99,80 %	n.d.	99,89 %
	{XYYYYY}1		0,00 %		0,03 %
	{XYYYYY}2		0,16 %		0,03 %
	{XYYYYY}3		0,04 %		0,00 %
	{XYYYYY}4		0,00 %		0,01 %
	{YYYYYY}5		0,00 %		0,00 %
Leu-233	{111111}		0,00 %		0,04 %
Lys-431	{000000}	n.d.	99,77 %	n.d.	99,74 %
	{YYYYYY}1		0,00 %		0,00 %
	{YYYYYY}2		0,00 %		0,00 %
	{YYYYYY}3		0,23 %		0,26 %
	{YYYYYY}4		0,00 %		0,00 %
	{YYYYYY}5		0,00 %		0,00 %
	{111111}		0,00 %		0,00 %

		J774A.1 (I)	*L.m.* (I)	J774A.1(II)	*L.m.* (II)
		**2 mM [U-$^{13}C_5$]glutamine**			
		**(-IFN-γ / infected)**			
Phe-336	{000000000}	n.d.	99,84 %	n.d.	99,82 %
	{YYYYYYYYY}1		0,04 %		0,00 %
	{YYYYYYYYY}2		0,00 %		0,00 %
	{YYYYYYYYY}3		0,00 %		0,00 %
	{YYYYYYYYY}4		0,11 %		0,15 %
	{YYYYYYYYY}5		0,01 %		0,02 %
	{YYYYYYYYY}6		0,00 %		0,00 %
	{YYYYYYYYY}7		0,00 %		0,01 %
	{YYYYYYYYY}8		0,00 %		0,00 %
	{111111111}		0,00 %		0,00 %
Pro-286	{00000}	n.d.	99,89 %	n.d.	99,85 %
	{YYYYY}1		0,00 %		0,00 %
	{YYYYY}2		0,00 %		0,00 %
	{YYYYY}3		0,03 %		0,00 %
	{YYYYY}4		0,00 %		0,01 %
	{11111}		0,09 %		0,13 %
Ser-390	{000}	99,86 % ± 0,03 %	99,98 %	99,63 % ± 0,22 %	99,96 %
	{YYY}1	0,00 % ± 0,00 %	0,02 %	0,29 % ± 0,27 %	0,00 %
	{YYY}2	0,00 % ± 0,00 %	0,00 %	0,00 % ± 0,00 %	0,00 %
	{111}	0,14 % ± 0,03 %	0,00 %	0,08 % ± 0,06 %	0,04 %
Thr-404	{0000}	n.d.	97,77 %	n.d.	98,80 %
	{YYYY}1		1,43 %		0,61 %
	{YYYY}2		0,80 %		0,59 %
	{YYYY}3		0,00 %		0,00 %
	{1111}		0,00 %		0,00 %
Tyr-466	{000000000}	n.d.	99,65 %	n.d.	99,55 %
	{YYYYYYYYY}1		0,01 %		0,00 %
	{YYYYYYYYY}2		0,15 %		0,31 %
	{YYYYYYYYY}3		0,07 %		0,00 %
	{YYYYYYYYY}4		0,00 %		0,00 %
	{YYYYYYYYY}5		0,10 %		0,10 %
	{YYYYYYYYY}6		0,00 %		0,00 %
	{YYYYYYYYY}7		0,00 %		0,00 %
	{YYYYYYYYY}8		0,01 %		0,00 %
	{111111111}		0,00 %		0,05 %
Val-288	{00000}	n.d.	99,98 %	n.d.	99,98 %
	{YYYYY}1		0,00 %		0,00 %
	{YYYYY}2		0,00 %		0,00 %
	{YYYYY}3		0,01 %		0,01 %
	{YYYYY}4		0,01 %		0,00 %
	{11111}		0,00 %		0,00 %

		2 mM [U-$^{13}C_5$]glutamine			
		(+IFN-γ / infected)			
		J774A.1 (I)	*L.m.* (I)	J774A.1 (II)	*L.m.* (II)
Ala-260	{000}	99,63 % ± 0,03 %	99,99 %	99,73 % ± 0,02 %	99,96 %
	{YYY}1	0,00 % ± 0,00 %	0,00 %	0,00 % ± 0,00 %	0,00 %
	{YYY}2	0,07 % ± 0,03 %	0,01 %	0,02 % ± 0,02 %	0,04 %
	{111}	0,30 % ± 0,01 %	0,00 %	0,26 % ± 0,01 %	0,00 %
Asp-418	{0000}	88,65 % ± 0,17 %	99,65 %	91,61 % ± 0,09 %	100,00 %
	{YYYY}1	0,10 % ± 0,09 %	0,35 %	0,11 % ± 0,09 %	0,00 %
	{YYYY}2	1,15 % ± 0,02 %	0,00 %	0,58 % ± 0,04 %	0,00 %
	{YYYY}3	1,90 % ± 0,01 %	0,00 %	1,22 % ± 0,05 %	0,00 %
	{1111}	8,19 % ± 0,08 %	0,00 %	6,49 % ± 0,07 %	0,00 %
Glu-432	{00000}	65,52 % ± 0,05 %	99,04 %	70,73 % ± 0,14 %	100,00 %
	{YYYYY}1	0,05 % ± 0,05 %	0,00 %	0,00 % ± 0,00 %	0,00 %
	{YYYYY}2	0,60 % ± 0,08 %	0,00 %	0,46 % ± 0,05 %	0,06 %
	{YYYYY}3	2,45 % ± 0,01 %	0,00 %	1,74 % ± 0,02 %	0,00 %
	{YYYYY}4	1,40 % ± 0,02 %	0,07 %	1,19 % ± 0,04 %	0,00 %
	{11111}	29,97 % ± 0,06 %	0,89 %	25,89 % ± 0,12 %	0,00 %
Gly-246	{00}	99,80 % ± 0,06 %	100,00 %	99,84 % ± 0,05 %	100,00 %
	{YY}1	0,16 % ± 0,05 %	0,00 %	0,13 % ± 0,04 %	0,00 %
	{11}	0,03 % ± 0,02 %	0,00 %	0,03 % ± 0,03 %	0,00 %
His-440	{000000}	n.d.	99,84 %	n.d.	98,93 %
	{YYYYYY}1		0,00 %		0,00 %
	{YYYYYY}2		0,00 %		0,11 %
	{YYYYYY}3		0,02 %		0,48 %
	{YYYYYY}4		0,04 %		0,18 %
	{YYYYYY}5		0,06 %		0,13 %
	{111111}		0,03 %		0,18 %
Ile-200	{X00000}	n.d.	99,08 %	n.d.	97,54 %
	{XYYYYY}1		0,53 %		0,89 %
	{XYYYYY}2		0,04 %		0,89 %
	{XYYYYY}3		0,00 %		0,01 %
	{XYYYYY}4		0,00 %		0,00 %
	{YYYYYY}5		0,00 %		0,15 %
Ile 233	{111111}		0,35 %		0,53 %
Leu-274	{X00000}	n.d.	99,92 %	n.d.	99,82 %
	{XYYYYY}1		0,00 %		0,00 %
	{XYYYYY}2		0,06 %		0,14 %
	{XYYYYY}3		0,02 %		0,04 %
	{XYYYYY}4		0,00 %		0,00 %
	{X11111}		0,00 %		0,00 %
Leu-233	M+6		0,00 %		0,00 %
Lys-431	{000000}	n.d.	99,79 %	n.d.	99,76 %
	{YYYYYY}1		0,00 %		0,00 %
	{YYYYYY}2		0,00 %		0,00 %
	{YYYYYY}3		0,21 %		0,24 %
	{YYYYYY}4		0,00 %		0,00 %
	{YYYYYY}5		0,00 %		0,00 %
	{111111}		0,00 %		0,00 %

		2 mM [U-$^{13}C_5$]glutamine			
		(+IFN-γ / infected)			
		**J774A.1 (I)**	***L.m.* (I)**	**J774A.1 (II)**	***L.m.* (II)**
Phe-336	{000000000}	n.d.	99,77 %	n.d.	99,86 %
	{YYYYYYYYY}1		0,08 %		0,00 %
	{YYYYYYYYY}2		0,00 %		0,00 %
	{YYYYYYYYY}3		0,00 %		0,00 %
	{YYYYYYYYY}4		0,11 %		0,12 %
	{YYYYYYYYY}5		0,00 %		0,01 %
	{YYYYYYYYY}6		0,03 %		0,01 %
	{YYYYYYYYY}7		0,00 %		0,00 %
	{YYYYYYYYY}8		0,00 %		0,00 %
	{111111111}		0,00 %		0,00 %
Pro-286	{00000}	n.d.	99,98 %	n.d.	99,96 %
	{YYYYY}1		0,01 %		0,00 %
	{YYYYY}2		0,00 %		0,00 %
	{YYYYY}3		0,00 %		0,00 %
	{YYYYY}4		0,00 %		0,03 %
	{11111}		0,01 %		0,01 %
Ser-390	{000}	99,90 % ± 0,03 %	99,93 %	99,79 % ± 0,11 %	99,65 %
	{YYY}1	0,00 % ± 0,00 %	0,07 %	0,12 % ± 0,11 %	0,27 %
	{YYY}2	0,00 % ± 0,00 %	0,00 %	0,00 % ± 0,00 %	0,05 %
	{111}	0,10 % ± 0,03 %	0,00 %	0,09 % ± 0,01 %	0,03 %
Thr-404	{0000}	n.d.	98,99 %	n.d.	98,24 %
	{YYYY}1		0,33 %		0,74 %
	{YYYY}2		0,68 %		1,01 %
	{YYYY}3		0,00 %		0,00 %
	{1111}		0,00 %		0,00 %
Tyr-466	{000000000}	n.d.	99,59 %	n.d.	99,56 %
	{YYYYYYYYY}1		0,00 %		0,04 %
	{YYYYYYYYY}2		0,27 %		0,22 %
	{YYYYYYYYY}3		0,00 %		0,08 %
	{YYYYYYYYY}4		0,00 %		0,00 %
	{YYYYYYYYY}5		0,08 %		0,08 %
	{YYYYYYYYY}6		0,03 %		0,00 %
	{YYYYYYYYY}7		0,00 %		0,00 %
	{YYYYYYYYY}8		0,02 %		0,00 %
	{111111111}		0,00 %		0,01 %
Val-288	{00000}	n.d.	99,96 %	n.d.	99,99 %
	{YYYYY}1		0,00 %		0,00 %
	{YYYYY}2		0,00 %		0,00 %
	{YYYYY}3		0,01 %		0,00 %
	{YYYYY}4		0,01 %		0,00 %
	{11111}		0,02 %		0,00 %
